# A multi-point heart rate monitoring using a soft wearable system based on fiber optic technology

**DOI:** 10.1038/s41598-021-00574-2

**Published:** 2021-10-27

**Authors:** Daniela Lo Presti, Francesca Santucci, Carlo Massaroni, Domenico Formica, Roberto Setola, Emiliano Schena

**Affiliations:** 1 Unit of Measurements and Biomedical Instrumentation, Departmental Faculty of Engineering, Università Campus Bio-Medico di Roma, Via Alvaro del Portillo, 21 00128 Rome, RM Italy; 2 Departmental Faculty of Engineering, Unit of Automatic Control, Università Campus Bio-Medico di Roma, Via Alvaro del Portillo, 21 00128 Rome, RM Italy; 3 Unit of NEXT, Departmental Faculty of Engineering, Università Campus Bio-Medico di Roma, Via Alvaro del Portillo, 21 00128 Rome, RM Italy

**Keywords:** Biomedical engineering, Optical sensors

## Abstract

Early diagnosis can be crucial to limit both the mortality and economic burden of cardiovascular diseases. Recent developments have focused on the continuous monitoring of cardiac activity for a prompt diagnosis. Nowadays, wearable devices are gaining broad interest for a continuous monitoring of the heart rate (HR). One of the most promising methods to estimate HR is the seismocardiography (SCG) which allows to record the thoracic vibrations with high non-invasiveness in out-of-laboratory settings. Despite significant progress on SCG, the current state-of-the-art lacks both information on standardized sensor positioning and optimization of wearables design. Here, we introduce a soft wearable system (SWS), whose novel design, based on a soft polymer matrix embedding an array of fiber Bragg gratings, provides a good adhesion to the body and enables the simultaneous recording of SCG signals from multiple measuring sites. The feasibility assessment on healthy volunteers revealed that the SWS is a suitable wearable solution for HR monitoring and its performance in HR estimation is strongly influenced by sensor positioning and improved by a multi-sensor configuration. These promising characteristics open the possibility of using the SWS in monitoring patients with cardiac pathologies in clinical (e.g., during cardiac magnetic resonance procedures) and everyday life settings.

## Introduction

Cardiovascular diseases (CVDs), primarily ischemic heart disease and stroke, are the leading cause of death, accounting for 31% of all global deaths^1^. In 2019, CVDs claimed 18.9 million lives worldwide^2^. Moreover, CVDs impose a serious economic burden on public health: in 2017 medical direct costs related to CVDs amounted to $318 billion, and they are expected to reach $1.1 trillion in 2035^3^. A prompt diagnosis is crucial to prevent premature deaths and achieve a significant decrease in the mortality rate and health care costs. For a timely detection of physiological warning signs, the continuous cardiac monitoring would be very helpful, allowing to avert cardiovascular emergencies that require rapid and resource-intensive care^4^. In particular, a consistent number of epidemiological studies have pointed out a strong association between elevated resting heart rate (HR) and an increased risk of cardiovascular morbidity^5–7^.

In recent years wearable devices have attracted significant attention in the scientific community for the evaluation of heart rhythm over extended periods of time in out-of-laboratory settings due to their characteristics of non-invasiveness, flexibility, and compatibility with the skin^8,9^. Innovations in wearable technologies have made it possible to record both the electric impulses^10,11^ (i.e., electrocardiography) and the precordial movements of the heart. Among various techniques, ballistocardiography-BCG (i.e., a measurement of the ballistic forces generated by the ejection of blood into the vessels) and seismocardiography-SCG^12^ (i.e., a recording of the chest wall vibrations in response to the atrioventricular contractions) are gaining broad interest for a non-invasive detection of the heart activity in everyday settings^13^, where traditional methods show their main limitations. Scientific evidence has been collected on the ability of the SCG to reflect cardiac mechanics and on the feasibility to estimate HR using SCG signal features. The SCG was first observed by Bozhenko in 1961^14^, and its first application in clinical studies was in 1991, by Salerno and Zanetti^15^. Initially, this technique was abandoned by the scientific community mainly due to the accelerometers cumbersome size, but the advent of micro-electro-mechanical systems led to miniaturized inertial measurement units (IMUs), which integrate accelerometers and gyroscopes^16^. As a consequence, gyrocardiography (GCG) has also been proposed as a new non-invasive technique for assessing cardiac activity by using the angular velocities yielded by the gyroscope^17^.

Despite the portable features and miniaturized size of IMUs, physical strapping is required to reduce the acoustic mismatch caused by the air gap between the rigid sensing unit and the human chest. To overcome these issues, recent technological advances in flexible materials have brought to the development of wearable strain sensors that mimic the skin stretchability and softness^18,19^, such as stretchable transistors and e-tattoos in direct contact with the chest^20–23^. Another attractive solution is represented by fiber Bragg grating sensors (FBGs) encapsulated into polymer matrices^24^. These optical strain sensors have unique metrological (e.g., high sensitivity, accuracy, low zero drift, multiplexing capability), geometrical (e.g., small size, light weight), and mechanical properties (e.g., flexibility, durability)^25^. Such distinctive features fostered the use of FBGs for structural health monitoring making this technology mature for applications in civil engineering and aerospace^26^ and in some medical applications (e.g., respiratory monitoring, minimally invasive surgery and biomechanics). However, moving towards SCG monitoring, the FBG technological readiness level comes down dramatically^25^. Only few studies investigated FBG-based systems for recording SCG signals from laying or seated subjects by wearable systems (e.g., smart garments) or instrumented objects (e.g., mattresses and chairs)^25,27,28^ showing low measurement reliability in HR monitoring^25^. The main limitation of these wearable solutions relies on their single-sensor configuration to record SCG signal from one measurement site^12^. Such a design does not allow to investigate the influence of sensor positioning on the SCG signal despite its well-known strong impact on waveform morphology, amplitude, and clinically relevant features^29–31^. For this reason, literature is still lacking standardized locations for reliable SCG records.

Here we present for the first time a skin-like multisensory wearable system based on FBG technology for HR monitoring from SCG signal (hereinafter called soft wearable system-SWS). The key features of the proposed system are the multi-sensor configuration based on an FBG array and its encapsulation into a skin-like soft matrix. The array configuration allows multi-point measurements of heart-induced vibrations with a reduced encumbrance and a high spatial resolution, while the use of a soft interface improves the robustness of the optical fiber, the FBGs adherence to a substrate, the system adaptability to the natural contours of the human body and the user acceptability. These distinctive features may promote scientific innovations in the SCG detection by addressing the need for: (i) improvements of HR measurement accuracy and reliability, (ii) knowledge advancements of the sensor locations influence on the SCG signal; (iii) definition of a promising sensor position on the chest surface for HR measurements, and (iv) improvements of FBG signal processing for HR estimation from SCG records.

## Results

### Soft wearable system: characteristics and testing setup

**Figure 1:**
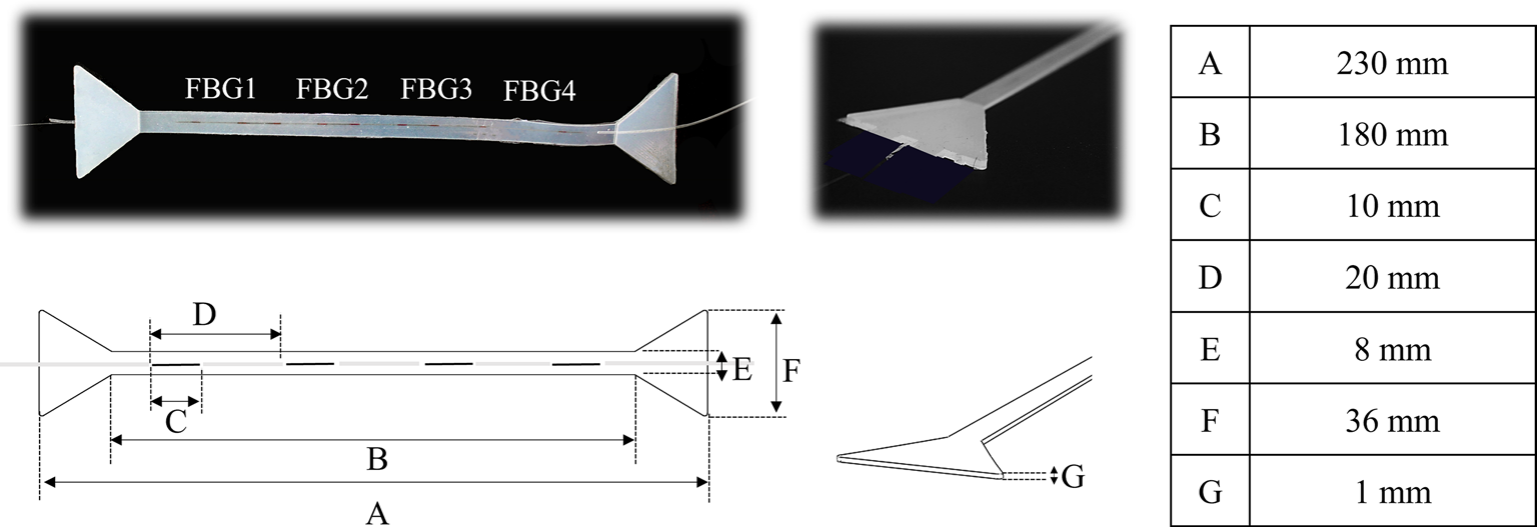
The soft skin like wearable system with the four FBGs encapsulated into: pictures and geometrical features.

The SWS consists of an array of four FBGs (i.e., FBG1, FBG2, FBG3, and FBG4) encapsulated into a flexible matrix of silicone rubber. These SWS features allow investigating a wider area on the chest wall surface, covering multiple promising measurement sites simultaneously. The overall dimensions of the flexible matrix are 230 mm × 36 mm × 1 mm with a narrow portion of 180 mm × 8 mm × 1 mm (see Fig. 1). The output of each FBG is an optical signal (i.e., the so-called Bragg wavelength, λ_B_) that undergoes a shift in accordance with strain and temperature variations^32^. Usually, the shift from the initial λ_B_ value is indicated as Δλ_B_ (see Sect. 1, Supplementary Material).

**Figure 2:**
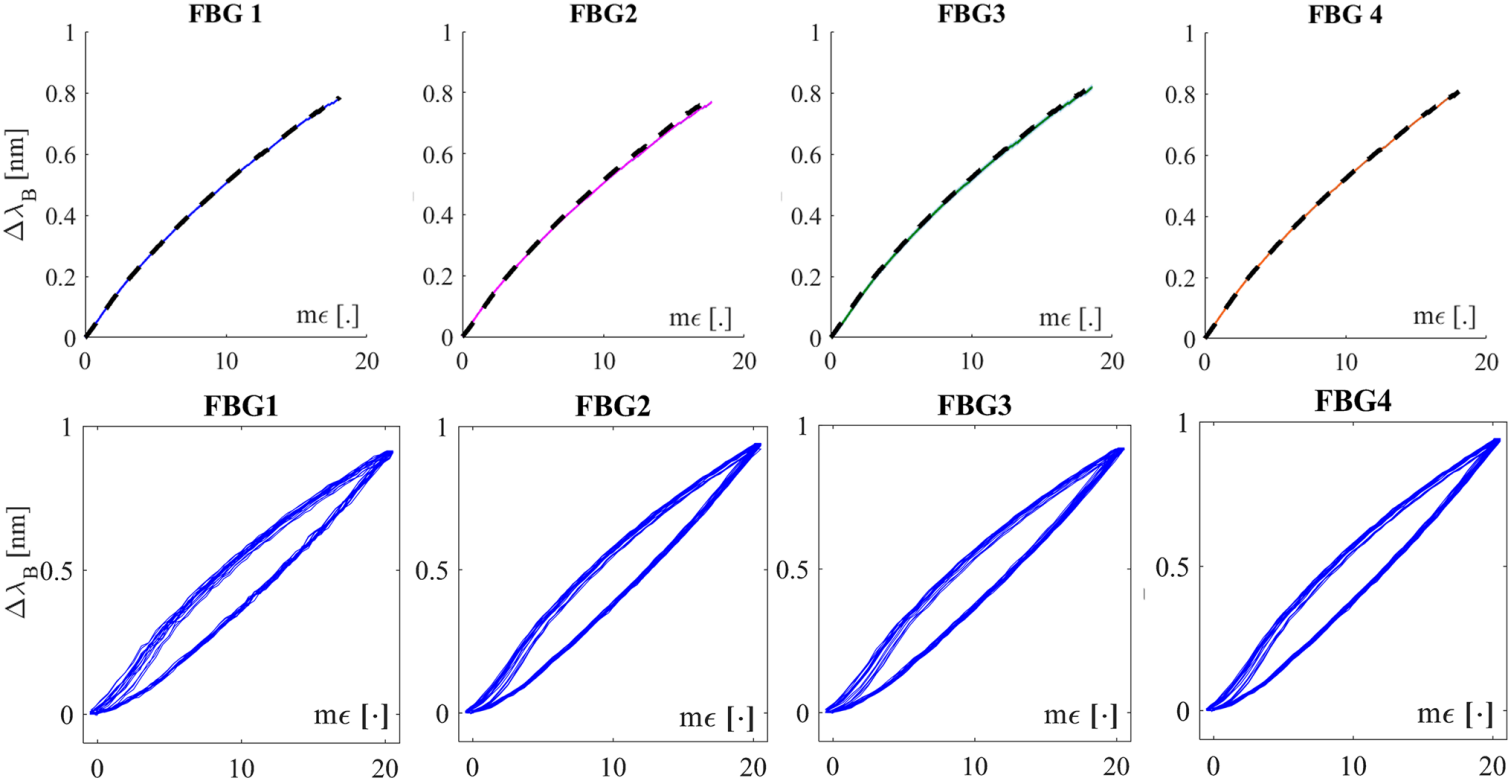
The calibration curves (experimental data with fitting curves in dotted line and shadowed uncertainty) and the hysteresis loops at velocities mimicking 60 bpm for FBG1, FBG2, FBG3, and FBG4.

The sensing mechanism is based on the strain (ε) transmission between the chest wall and the silicone matrix via mechanical coupling. A tight coupling is required considering that the heart-induced vibrations onto the chest surface are fast (from 1 to 1.34 Hz) and weak (amplitude ranging from 0.2 mm and 0.5 mm)^33^. Moreover, the multiplexing capability of the FBGs enables the simultaneous measurement of cardiac vibrations from multiple sites. The SWS metrological characteristics were evaluated before its feasibility assessment on healthy volunteers. All the FBGs showed a similar strain sensitivity (0.044 nm·με^−1^, 0.045 nm·mε^−1^, 0.045 nm·mε^−1^, 0.046 nm·mε^−1^ for FBG1, FBG2, FBG3, and FBG4, respectively) with acceptable hysteresis errors always ≤ 30.4% as shown in Fig. 2.

For further details see Supplementary Material (Sect. 2, Supplementary Figs. 3, 4, Tables 1, and 2).

The SWS was taped to an elastic T-shirt in three specific positions: (i) horizontally on the left sternum from the xiphoid process to the heart apex (from now on Position 1), (ii) vertically from the xiphoid process to the umbilicus (from now on Position 2), and (iii) horizontally from the right lumbar to the left lumbar region just above the umbilicus on the periumbilical area (from now on Position 3). All these locations were chosen since they are considered promising sites for SCG recording in the literature and easily identifiable by two well-recognizable anatomical landmarks (i.e., xiphoid process and umbilicus) on the frontal plane of the chest wall by both expert (e.g., clinicians) and inexpert users (e.g., patients). The ends of the silicone matrix were secured to the garment by using a medical tape. Electrocardiogram (ECG) was used as a benchmark. For further details, see Supplementary Material (Sect. 3, Supplementary Fig. 5).

All subjects enrolled in this study (i.e., S1, S2, S3, S4, S5, S6, and S7) were healthy with no history of cardiac disease (preclinical trial titled Smart Textile—Università Campus Bio-Medico di Roma, protocol number ST-UCBM 27.2(18)0.20 OSS granted by the Ethical Committee of Università Campus Bio-Medico di Roma, Rome, ITALY). The group has the following characteristics (expressed as mean ± standard deviation): age of 26 ± 1 years, height of 184 ± 12 cm, and body mass of 80.7 ± 8.1 kg with thoracic and abdominal circumferences of 98.2 ± 1.8 cm and 83.7 ± 6.8 cm, respectively.

Each subject performed the following test in a supine position:

1. The SWS was placed in Position 1 and the subject was invited to hold his breath for ~ 20 s. The outputs of the FBGs and the ECG sensor were synchronized using a trigger signal at the beginning of the apnea stage. The same test was repeated by placing the system in Position 2 and Position 3.
2. The same procedure was performed three times cyclically (i.e., tests: T1, T2, and T3). Hence, a total of nine tests were carried out for each subject, three tests per position.

**Figure 3:**
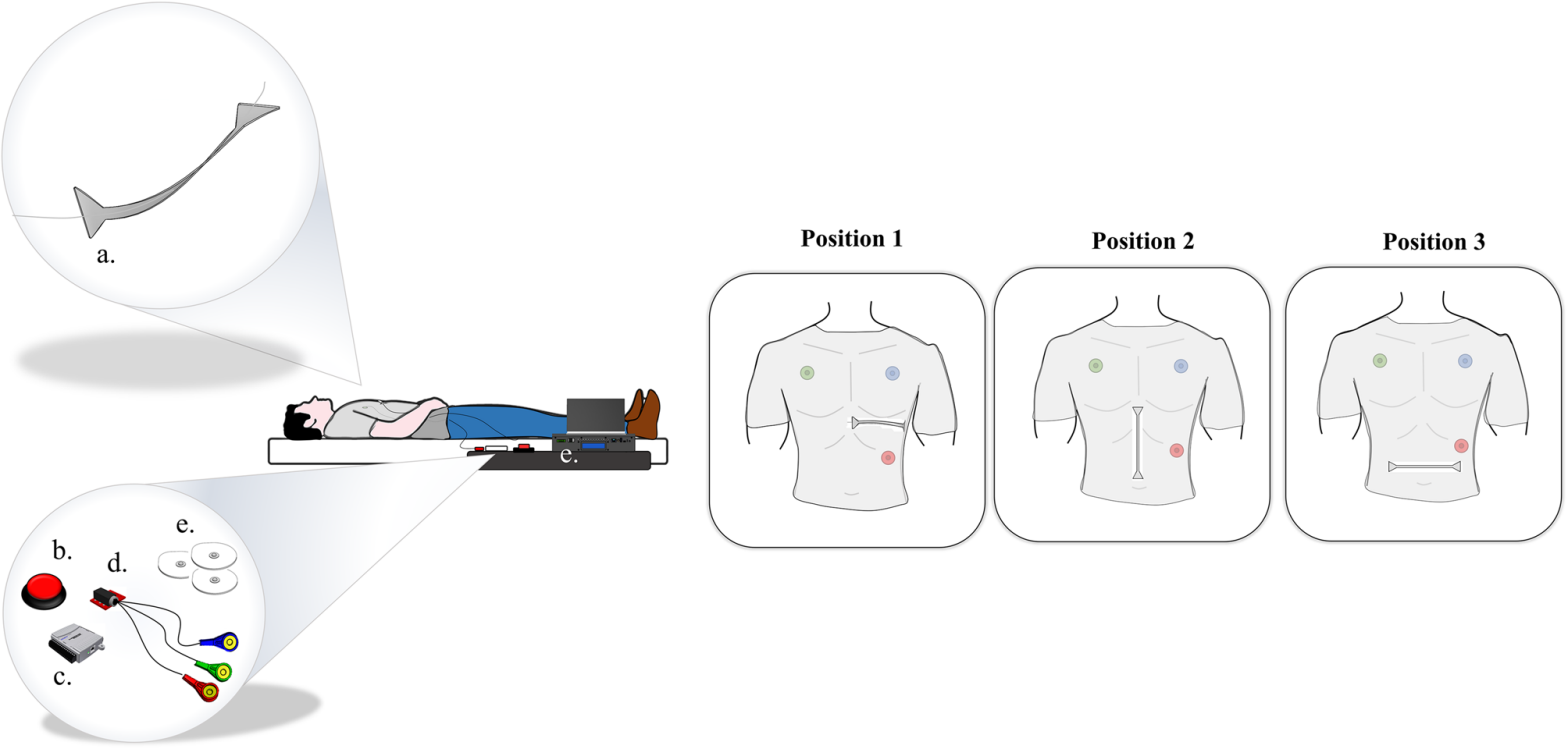
The experimental setup during the test procedure. The SWS (**a**) taped on the T-shirt, the push button for the signal synchronization (**b**), the DAQ (**c**) used to collect the ECG trace and the trigger signal, the ECG sensor (**d**) and the FBG interrogator (**e**).

During each test, the FBGs outputs, the ECG, and the trigger signal were simultaneously collected and showed in real time. The experimental setup is schematically reported in Fig. 3 (partially available from https://www.dimensions.com).

### Soft wearable system: feasibility assessment

**Figure 4:**
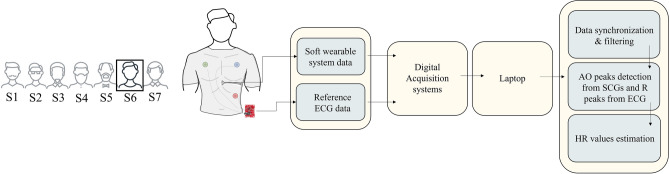
A block representation of the main steps of the data collection and processing to estimate HR.

The SWS was assessed considering 15 s of data recording during apnea. The output of the FBGs and the ECG sensor were passband filtered and used to estimate the mean HR values from the peaks detected over time. Figure 4 reports a block diagram with the main steps carried out for the data analysis: from data acquisition to HR estimation for each volunteer.

The estimation of HR by the SWS was performed considering the peaks related to the aortic valve opening (AO). This event was detected on the SCG envelope considering the output of each FBG (FBG1, FBG2, FBG3, FBG4) and their sum (i.e., FBGsum). The envelope is determined using the magnitude of its analytic signal computed by filtering the SCG signal with a Hilbert FIR filter. The reference HR values were estimated by identifying the R peaks of the ECG signal.

**Figure 5:**
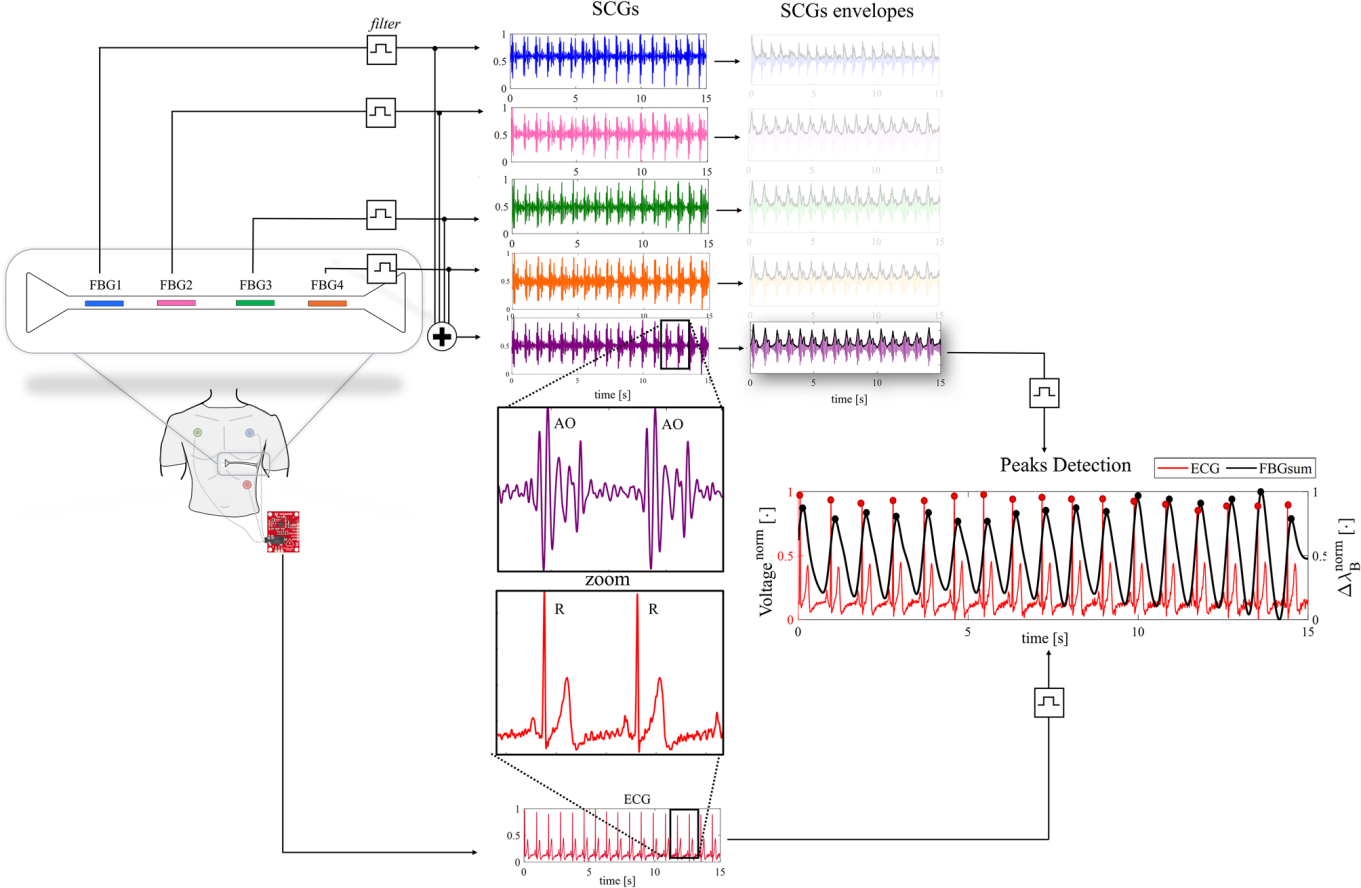
The main steps of the data processing to estimate HR. The SCG signals from the passband filtered FBG1 (blue), FBG2 (magenta), FBG3 (green), FBG4 (orange), and FBGsum (purple) and the ECG signal (red) are shown. The zoom on two consecutive peaks of SCG and ECG signals is reported, highlighting AO and R peaks. The SCG envelopes and the peak detection for a signal from the SWS (i.e., FBGsum) and the ECG is also shown.

The SCG signals from FBG1, FBG2, FBG3, FBG4, and FBGsum are shown in Fig. 5 with zooms on two consecutive cardiac cycles on an SCG signal (with AO peaks) and the corresponding ECG (with R peaks). The SCG envelopes and the final stage corresponding to the peaks detection on the SWS and the ECG are also shown in Fig. 5.

**Figure 6:**
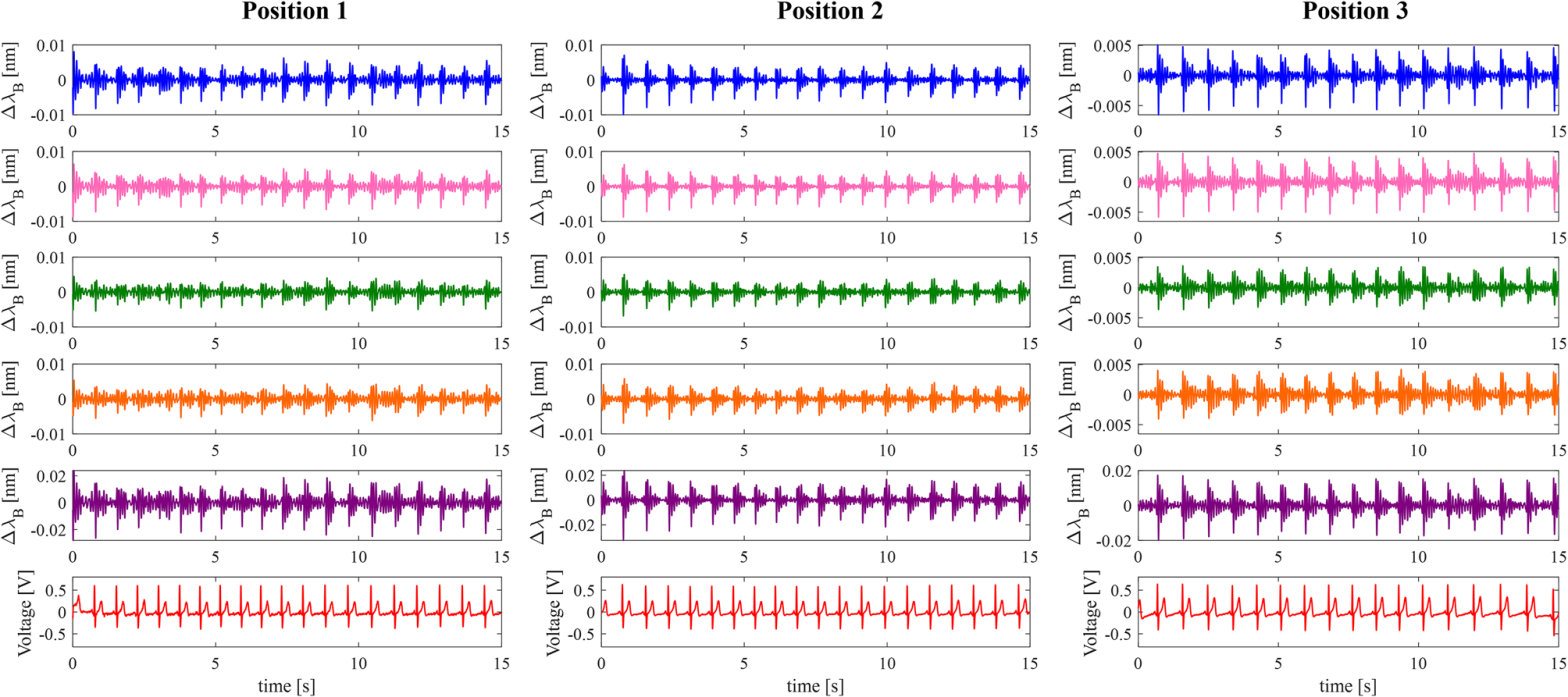
The SCGs from FBG1 (blue line), FBG2 (magenta line), FBG3 (green line), FBG4 (orange line), and FBGsum (purple line) and the ECG signal (red line) and from a single volunteer considering Position 1, Position 2 and Position 3 are shown.

A further description of this analysis is included in Supplementary Material (Sect. 4, Supplementary Figs. 6, 7, 8, 9). Figure 6 shows the SCG signals obtained for a volunteer when the SWS is placed in Position1, Position 2, and Position 3 including the reference ECG signal.

**Figure 7:**
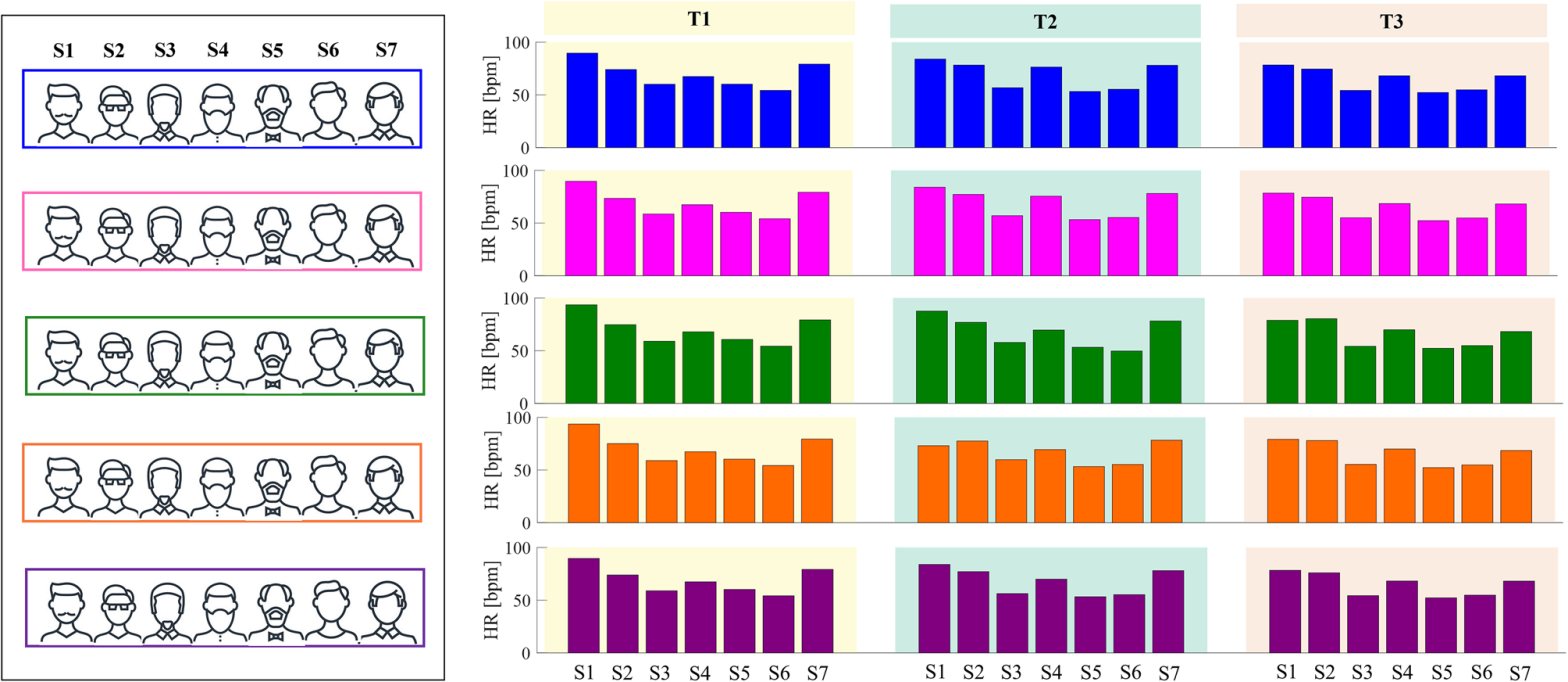
Schematic of the approach used to group the HR data considering a single position.

The HR values estimated by the SWS were grouped as follows: for each position, the mean HR values were estimated by FBG1 (blue), FBG2 (magenta), FBG3 (green), FBG4 (orange), FBGsum (purple) considering all the volunteers and all the tests (i.e., T1, T2 and T3). For a better understanding, Fig. 7 shows the HR values of all the subjects when the SWS is in Position 1. In each position, a total of 21 values were obtained for each FBG since seven volunteers performed three times the same protocol.

**Figure 8:**
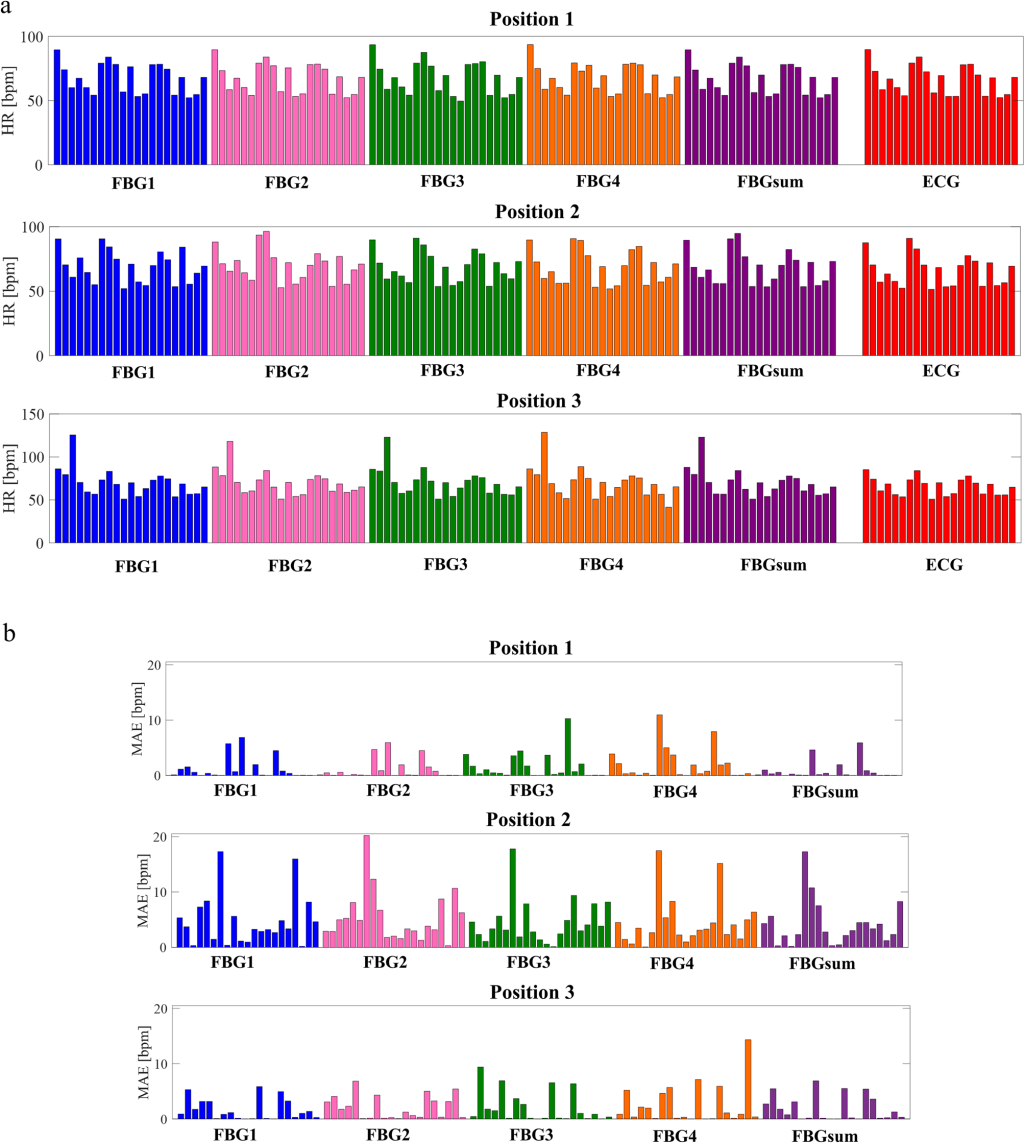
(**a**) The mean HR values obtained by the FBG1 (blue bars), FBG2 (magenta bars), FBG3 (green bars), FBG4 (orange bars), FBGsum (purple bars) considering the three positions, the three tests and the seven subjects. The reference values estimated by the ECG sensor (red bars) are also reported. (**b**) The MAE values obtained comparing each HR value estimated by the SWS considering FBG1, FBG2, FBG3, FBG4, and FBGsum with the reference one.

Once HR values have been estimated, the mean absolute error (MAE) was calculated to perform a quantitative assessment of the agreement between the proposed system and the reference one in both the aforementioned analyses. Figure 8a summarizes all the HR values estimated by FBG1 (blue bars), FBG2 (magenta bars), FBG3 (green bars), FBG4 (orange bars), FBGsum (purple bars) and ECG sensor (red bars). Figure 8b shows the MAE values grouped per position. Once the MAE values have been estimated, two analyses were carried out to investigate the influence of the SWS positioning and multi-sensor configuration on the system performance.

The first analysis (i.e., Position analysis) aimed at assessing the performance of the SWS to underline in which position the proposed device shows the highest accuracy in HR monitoring. The second one (i.e., Single-sensor vs. Multi-sensor analysis) was carried out to compare results from FBG1, FBG2, FBG3, FBG4 with the ones from FBGsum to find out if the multi-sensor configuration improves the HR measurement accuracy.

### Position analysis

The HR values measured by the SWS and the reference instrument were compared considering the single FBGs output (i.e., FBG1, FBG2, FBG3, FBG4) and their sum (i.e., FBGsum). Results showed that the lowest MAE values were found in Position 1 (1.20 bpm, 2.10 bpm, 1.67 bpm, 2.04 bpm and 0.81 bpm for FBG1, FBG2, FBG3, FBG4, and FBGsum, respectively), followed by Position 3 (MAE of 1.68 bpm, 2.13 bpm, 2.11 bpm, 2.57 bpm, and 1.89 bpm), and Position 2 (MAE of 3.22 bpm, 4.17 bpm, 2.85 bpm, 2,63 bpm, and 2.08 bpm). These results suggest that the SWS works better when placed horizontally (i.e., Position 1 and Position 3), than vertically (Position 2) and that the best performance is reached when the SWS is placed closer to the heart from the xiphoid process to the heart apex (i.e., Position 1).

### Single-sensor versus multi-sensor analysis

The performance of the SWS was evaluated through a comparative analysis between the single-sensor and the multi-sensor approach. This investigation was carried out by comparing: (i) the MAE of each FBG and their sum in Position 1, Position 2, and Position 3; (ii) the MAE of each FBG and their sum by averaging the results from all the positions for a comprehensive evaluation of the SWS performance. In the first case, the HR provided by the FBGsum showed a better performance than each FBG sensor considered individually. The second investigation reinforces this finding as shown in detail in the Supplementary Material (Sect. 4 - Supplementary Table 5). These results demonstrate that the multi-sensor approach obtained by summing the outputs of multiple sensors improves the SWS performance in HR estimation.

## Discussion

This study presented the design, development, and assessment of a SWS for HR monitoring. For the first time a skin-like multisensory system based on FBG technology has been proposed for HR monitoring via SCG signal. The distinctive features of the SWS can play a key role in promoting scientific innovations addressing crucial open challenges related to conventional sensors for SCG monitoring. The array of FBGs and its encapsulation inside a skin-like flexible matrix allows simultaneous SCG measurements from multiple measuring sites, an easy installation on a T-shirt and a tight coupling with the natural contours of the body. Such characteristics led to a strong technology acceptance, high accuracy in HR measurement, and the definition of a promising position for SCG monitoring (i.e., Position 1).

To date, different methods have been proposed for HR monitoring from SCG signal and the accelerometers are still the most used^16,34,35^. The majority of works proposed single-sensor configuration typically placed on the torso at the level of the xyphoid process^13^. Recently, the interest around the development of skin-like systems have fostered the use of flexible polymer matrices to encapsulate miniaturized sensing elements^36,37^ and reduce sensors slippages^38^. However, these wearables are mainly based on a single-sensor configuration that does not promote any scientific advancement in defining standardized locations for reliable SCG measurements.

There are two main limitations with the proposed study. First, the small and homogenous healthy population enrolled in this work. Although they provide a good initial proof of concept, further investigations will focus on increasing the sample size. The second limitation is the supine position assumed by the volunteers during the apnea stage to accurately investigate the sensor functioning when mainly subjected to heart pumping-related deformations and motion artifact minimization. This setup can be available in ambulatory conditions (e.g., on bedridden patients or during MRI examinations) or in daily environments when lying down (e.g., TV watching or sleeping). In addition, the multi-sensor configuration may be useful for the motion artifacts removal as shown in^36,39^ for dual sensor systems based on e-tattoo and IMU technology, respectively. Indeed, the combination of multiple outputs can improve results of digital processing especially when the motion data falls within the SCG frequency band. While better assessment of system functioning during motion is still needed, this study opens the possibility of monitoring the mechanical activity of the heart by detecting reliable SCG signals using a skin-like unobtrusive device.

From a clinical point of view, the proposed system could be used on high-risk patients to detect valves-induced abnormal flow during cardiac MRI^40^ (exploiting the FBG immunity to electromagnetic interferences) and to evaluate cardiac valves functioning after replacement^41^. This could support development of clinical tests for the identification of valve disorders and pathological characteristics by using SCG signals detected in conjunction with MRI. The SCG will provide quantitative measurements indicative of potential abnormalities in heart activity that require follow-up while MRI will offer images and information of the actual cardiovascular condition and related outcomes.

## Methods

### Strain sensor fabrication

Fabrication of the soft skin-like system required the encapsulation of a fiber optic array into a flexible matrix. The optical strain sensor is a commercially available FBG array (AtGrating Technologies, China) composed of four 10 mm-length sensing elements uniformly edge-to-edge spaced by 20 mm and inscribed into an Acrylate SMF-28e fiber. The FBGs multiplexed into the optical array have the following central wavelengths of the reflected spectrum: λ_B_ values of 1525 nm, 1533 nm, 1541 nm, 1549 nm for FBG1, FBG2, FBG3, FBG4, respectively. These wavelength values were chosen to avoid signal output overlapping even under maximum strain conditions during the application of interest. The system configuration (i.e., the number of encapsulated FBGs and the matrix shape) was ad-hoc designed to investigate a large area of the chest wall surface, covering multiple promising measurement sites simultaneously. The silicone compound used for the array encapsulation is the Dragon skin™ 20 rubber (Smooth On, USA), which is a bicomponent silicones mixed 1A:1B by weight or volume. The polymer synthetization started by mixing the liquid components A and B; then the mixture was thinned with the Silicone Thinner™ and vacuum degassed to minimize the air bubbles in the matrix. The FBG array was placed at the centre of the mould and the silicone poured into. Rubber cures at room temperature and once the rubber is vulcanized (after 4 h), the soft system was removed from the mould. The mould was custom designed using a 3D Software CAD (Solidworks® 2019) and 3D-printed using PLA to impress a dog-bone shape of 1 mm of thickness to the flexible matrix and encapsulate the sensing elements in the narrow portion to boosts stress in the central area of the matrix. As a result, an optimization of the system performances in terms of strain measurement can be achieved.

### Hardware setup

To feasibility assess the performance of the SWS, the FBGs array was tightly fixed to T-shirt using a medical adhesive tape and an ECG sensor (AD8232, SparkFun Electronics) was used as reference instrument. The AD8232 chip was connected to the DAQ (6009, National Instrument) and the ECG trace was displayed using a custom Virtual Instrument developed in LabVIEW 2017 environment, at the sampling frequency of 1 kHz. An FBG interrogator (si255 based on HYPERION platform; Micron Optics Inc.) was used to record the output of the FBGs inscribed into the array at a sampling rate of 1 kHz. A patch cord with an FC/APC connector was used to connect the FBGs outputs to the optical interrogator. Their trends were showed in real time using ENLIGTH sensing analysis software (Micron Optics). To synchronize the FBGs and ECG output, a push button with a pull-up resistor was connected to the DAQ and a trigger signal acquired at 1 kHz and displayed in LabVIEW environment. The acquisition of the trigger signal was enabled by connecting the terminals of a push button to the DAQ and the same LabVIEW interface developed for displaying the ECG trace was used to show the toggle generated when the button is pressed. Data were processed off-line in MATLAB R2020b.

### Testing procedure

In this study seven subjects, who never suffered from any kind of cardiovascular disease, were enrolled. The protocol was approved by the Local Ethics Medical Committee, Rome, ITALY (ST-UCBM 27.2(18)0.20 OSS) and informed consent was obtained from all the participants. All subjects voluntarily agreed to participate to the experimental trial and did not receive a monetary compensation. Age and anthropometric measures (i.e., height, body mass, and thoracic and abdominal circumferences) of each participant were collected before starting the experimental test procedure. Participants were asked to perform tests in accordance with relevant guidelines and regulations of the ST UCBM 27/18 OSS. Ech participant was invited to lay in a supine position with their arms outstretched along the body. The soft system was placed in three positions: Position 1 by placing SWS horizontally in correspondence of the left side of the sternum from the xiphoid process to the heart apex; Position 2 vertically from the xyphoid process to the umbilicus; Position 3 horizontally from the right to the left lumbar region just above the umbilicus. Each of the seven volunteers instructed to hold the breath for 20 s after a lung inflation at their maximum inspirational volume. When the apnea starts, the push button is pressed close to the SWS, so that the trigger signal was recorded and used for the synchronization of FBGs output changes with the ECG tracing. Each subject performed this procedure three times (T1, T2, and T3), each time with SWS in a different position: firstly, data acquisition is performed with SWS in Position 1, once the subject has accomplished task, SWS is moved to Position 2 and then to Position 3, in sequence.

### Data processing

MATLAB (Mathworks, R2020b) was used to process and analyze the data. Signals collected by the ECG sensor and the FBGs encapsulated into the soft system were passband filtered. The ECG signal was passband filtered between a lower cut-off frequency of 0.5 Hz and a higher cut-off frequency of 100 Hz and to easily detected the R peaks. Signals from the four FBGs encapsulated into the flexible matrix were passband filtered by using a lower cut-off frequency of 10 Hz and a higher cut-off frequency of 30 Hz in order to extract the SCG waveform from each FBG signals. The identification of peaks related to AO was performed by calculating the SCG signal envelope using the *envelope* function in Matlab. The envelope is determined using the magnitude of its analytic signal. The analytic signal is computed by filtering the SCG signal with a Hilbert FIR filter. Upper SCG envelopes were obtained and then, passband filtered between a lower cut-off frequency of 0.5 Hz and a higher cut-off frequency of 2 Hz.

The peaks detection was performed via *findpeaks* function using as input parameters: the normalized signal (either filtered ECG or SCG envelopes), the mean of the signal as “MinPeakHeight” and a number of samples proportional the dominant frequency of the power spectral density (PSD) as “MinPeakDistance”. The PSD was performed for both ECG and FBG1, FBG2, FBG3, FBG4, and FBGsum using *pwelch* function. Afterwards, the 70% of this frequency was multiplied to the sampling frequency (i.e., 1 kHz) and the result was used to set the “MinPeakDistance” in *findpeaks*. The beat-to-beat HR values were calculated over 15 s of the ECG signal and the SCG envelope and then, the average HR value over each window was computed. The capability of the soft skin-like wearable system of monitoring HR was assessed in terms of MAE. The MAE values represent the discrepancy in bpm between the average HR obtained from the SCG envelope of FBG1, FBG2, FBG3, FBG4, and FBGsum, and the average HR reference value obtained from the ECG signal. The feasibility assessment of the proposed sensing solution was performed carrying out three analyses. Firstly, an intra-FBG intra-position analysis was carried out to highlight the performance of each FBG according to the soft sensor position and then, an inter-FBG intra-position analysis was performed to highlight the FBG with the best performance when the wearable system holds the same position and the intra-FBG inter position analysis to underline the FBG with the best results aside from the soft sensor position.

### Ethical approval

This study was conducted in accordance with the Declaration of Helsinki and was approved by the Ethical Committee of Università Campus Bio-Medico di Roma, Rome, ITALY (ST-UCBM 27.2(18)0.20 OSS). The subjects were informed about the measurements and protocol procedure and provided their informed consent before participation.

## Supplementary Information

Supplementary Information.

## Data Availability

The data sets generated during and/or analyzed during the present study are available from the corresponding author upon reasonable request.
