# Supplementary material for: A multi-point heart rate monitoring using a soft wearable system based on fiber optic technology
Source: Sci Rep. 2021 Oct 27;11:21162. doi: 10.1038/s41598-021-00574-2 (PMC8551187; doi:10.1038/s41598-021-00574-2)
Supplement: Supplementary file 1 — Supplementary Information. [file 41598_2021_574_MOESM1_ESM.docx]

**Supplementary Material**

*“A multi-point cardiac monitoring using a flexible wearable system based on fiber optic technology”*

Daniela Lo Presti^1^, Francesca Santucci^2^, Carlo Massaroni^1^, Domenico Formica^3^, Roberto Setola^2^, Emiliano Schena^1^

^1^Unit of Measurements and Biomedical Instrumentation, Departmental Faculty of Engineering, Università Campus Bio-Medico di Roma, Via Alvaro del Portillo, 21 00128 Rome (RM)

^2^Unit of Automatic Control, Departmental Faculty of Engineering, Università Campus Bio-Medico di Roma, Via Alvaro del Portillo, 21 00128 Rome (RM)

^3^Unit of NEXT, Departmental Faculty of Engineering, Università Campus Bio-Medico di Roma, Via Alvaro del Portillo, 21 00128 Rome (RM)

Index

[1. Sensor Fabrication and Working Principle 3](#_Toc71206529)

[2. Sensor Metrological Characterization 5](#_Toc71206530)

[3. Sensor Placement 9](#_Toc71206531)

[4. Data analysis and Results 10](#_Toc71206532)

# Sensor Fabrication and Working Principle

The four main steps performed to develop the soft sensor are shown in the following figure (partially created in Solidworks 2019 SP03 Version 27.3.0.52, https://www.solidworks.com).


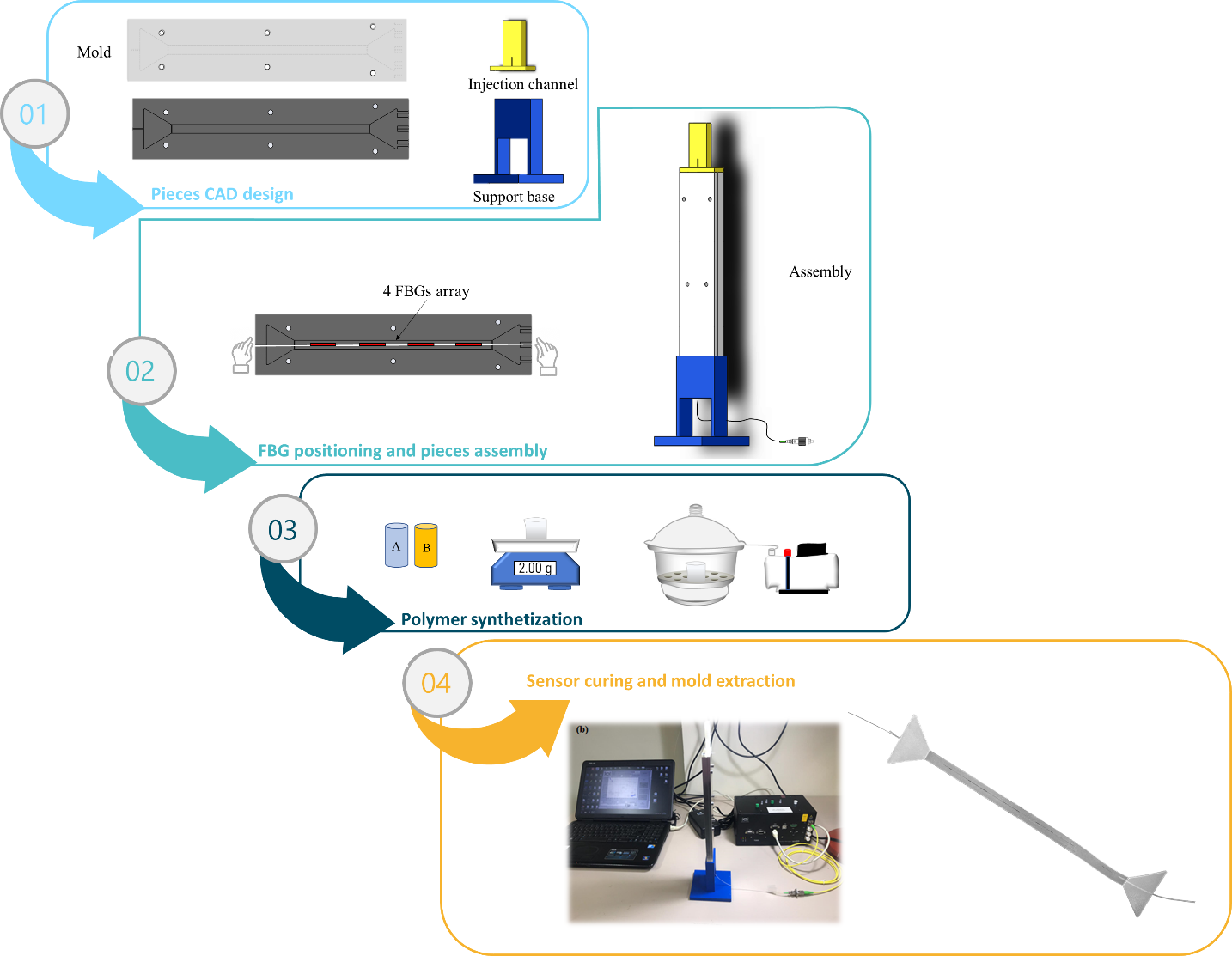


**Supplementary Figure 1**. The main fabrication steps: 01: mold design with complementary pieces necessary to fabricate the flexible sensor; 02: FBG array position inside the mold and mold assembly to allow the mold vertical filling using the injection channel, 03:polymer fabrication from A and B compounds mixing to the air bubble removal, 04: curing process (4h) and sensor extraction from the mold.

During fabrication step #02, the reflected spectrum of the FBG array placed inside the mold was collected as shown in the plot Intensity (I) vs. light wavelength (λ), see Supplementary Figure 2. The nominal values of λ_B_ are 1525.5 nm, 1533 nm, 1541 nm, 1549 nm for FBG1, FBG2, FBG3, and FBG4, respectively. During fabrication step #04, the FBGs outputs have been recorded and plotted in terms of Δλ_B_ vs. time.


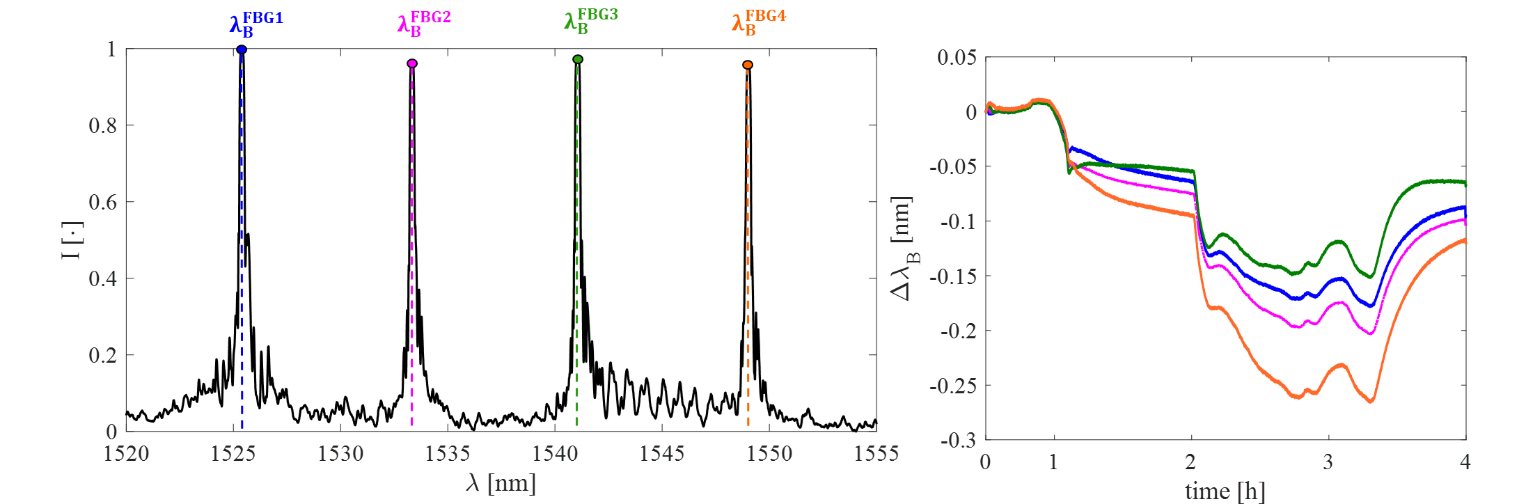


**Supplementary Figure 2**. The spectrum of the FBG array and the output changes during the curing process for FBG1 (blue line), FBG2 (magenta line), FBG3 (green line) and FBG4 (orange line), respectively.

After the curing process the soft wearable system is ready to be used. By exploiting the FBG intrinsic sensitivity to strain, the proposed system allows to detect the heart-induced vibration/deformation originated on the chest surface and to estimate HR from the SCG signal. An FBG is an optical strain gauge that works as a mirror: when a broadband spectrum of light goes inside the fiber and hits the inscribed grating a narrowband spectrum of light is back-reflected. The central wavelength of the reflected component satisfies the Bragg relation:

λ_B_ = 2η_eff_Λ

with η_eff_ the effective refractive index of the fiber core and Λ the grating period. The temperature and strain dependence of η_eff_ and Λ induces changes of λ_B_ (Δλ_B_) as function of temperature and/or strain. When incapsulated into a polymer matrix as a silicone rubber, the grating is shield by environmental parameters as (temperature and humidity changes) and when non in direct contact with the skin (as in the present study) also the influence of body temperature can be considered negligible.

Thus, the four FBG sensors encapsuled into the flexible matrix work as strain sensor: when the heart bits, small vibrations/deformations originate on the chest surface and strain the soft matrix. These strains are transduced to the grating causing cardiac-induced Δλ_B_.

# Sensor Metrological Characterization

- 1. Sensitivity to strain

The metrological characterization of the proposed system was performed to retrieve the sensitivity to strain (S_ε_) by means of a tensile testing machine (Instron, mod. 3365), at ambient temperature. The static assessment of the wearable system was carried out by positioning the sample between the lower and upper clamps of the machine. Quasi-static conditions were guaranteed by a low displacement rate (i.e., 2 mm· min^−1^) with respect to the system dynamic response. To cover the ε range that could be experienced by the sensor in response to heart rate, a maximum strain ($\varepsilon_{\max} \%$) of about 2 % (i.e., 4 mm) was used.

Indeed, the pumping action of the heart causes on the chest wall surface microscopic vibrations of amplitude 0.2 mm $\div$ 0.5 mm, in a frequency range 1 Hz – 1.34 Hz. The displacement applied by the tensile machine on the polymeric matrix during the trial was recorded at the sampling frequency of 10 Hz, while the $\Delta$λ_B_ values of the four FBGs were collected by the optical spectrum interrogator (si255 based on HYPERION platform; Micron Optics Inc.) at the sampling frequency of 100 Hz. This mechanical test was performed five times to investigate the repeatability of the system response to the applied ε. The raw data were processed through a custom algorithm to extract the calibration curve ($\Delta$λ_B_ vs. ε).


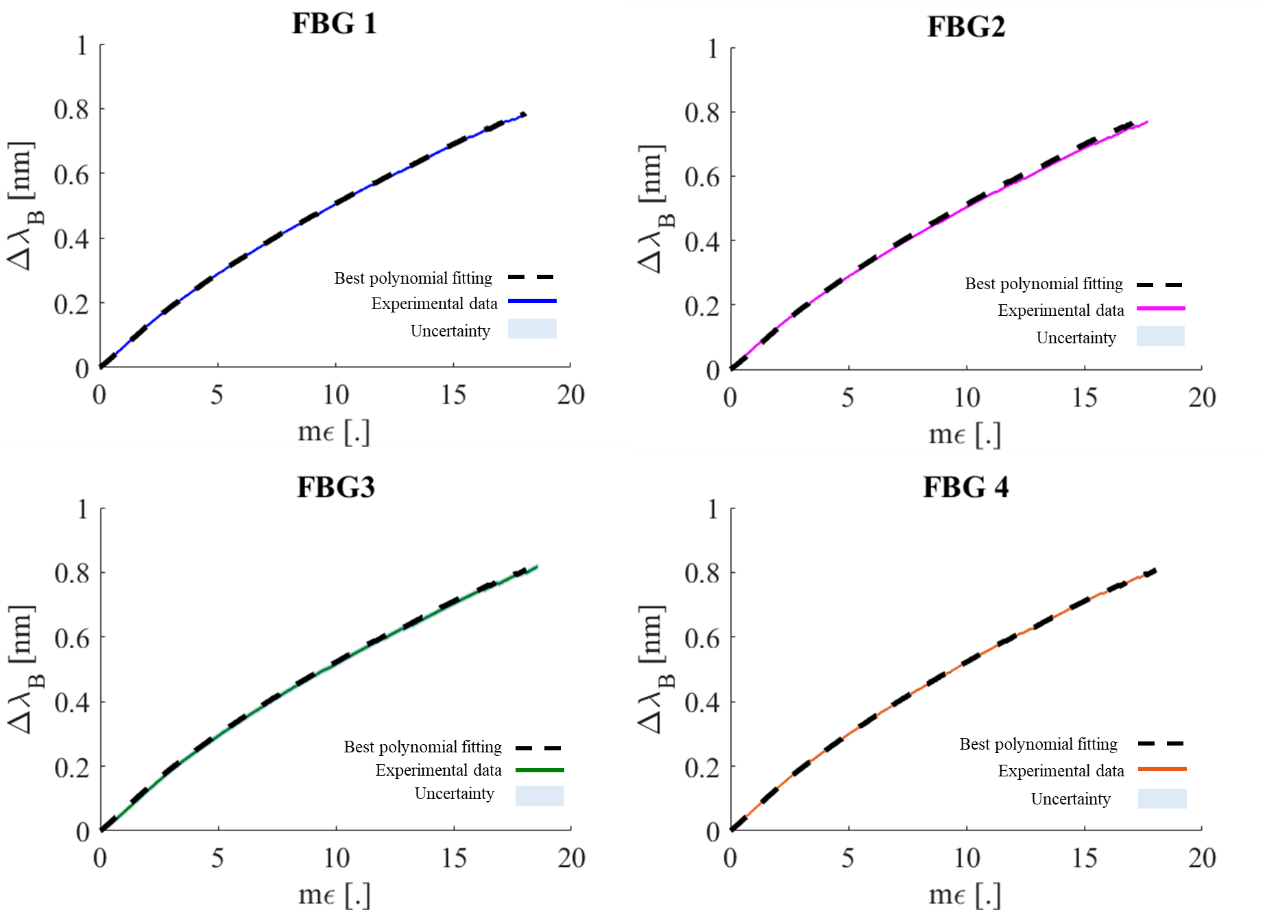


**Supplementary Figure 3**. The calibration curve with the best fitting and the related uncertainty for FBG1 (blue line), FBG2 (magenta line), FBG3 (green line) and FBG4 (orange line).

For each FBG, the mean value of $\Delta$λ_B_ and the expanded uncertainty were calculated across the five tests. The expanded uncertainty was obtained as the standard uncertainty multiplied by the coverage factor (k = 2.776), considering a t-student distribution with four degrees of freedom and a confidence level of 95%. The calibration curve was estimated as the best fitting polynomial curve (see Fig. 3). Data were fitted to a 2^nd^ order polynomial and the sensitivity to strain (S_ε_) was evaluated as:

$$S_{\varepsilon}= \frac{\Delta\lambda_{B}\left( {m\varepsilon}^{max} \right)- \Delta\lambda_{B}\left( {m\varepsilon}^{0} \right)}{{m\varepsilon}^{max}-{m\varepsilon}^{0}}$$

**Supplementary Table 1**. Calibration curves and S_ε_ values of the 4 FBGs. The variables of the calibration curves (i.e., y and x) represent the output of the sensor (i.e., $\Delta$λ_B_) and the strain (i.e., $m\varepsilon$)

| **Calibration curve** | | | |
| --- | --- | --- | --- |
| **FBG1** | **FBG 2** | **FBG 3** | **FBG 4** |
| $y=-0.0010\cdot x^{2}+0.0617\cdot x$ | $y=-0.0010\cdot x^{2}+0.0619\cdot x$ | $y=-0.0011\cdot x^{2}+0.0627\cdot x$ | $y=-0.0010\cdot x^{2}+0.0619\cdot x$ |
| **S_ε_ [nm/mε]** | | | |
| **FBG1** | **FBG 2** | **FBG 3** | **FBG 4** |
| 0.044 | 0.043 | 0.044 | 0.043 |

- 1. Hysteresis errors

Three dynamic tests consisting of 20 hysteresis cycles were performed at 3 different speeds (i.e., 240 mm·min^−1^, 480 mm·min^−1^and 720 mm·min^−1^), which allow simulating hear rate (HR) of 30 bpm, 60 bpm and 90 bpm, respectively. The outputs of the four FBGs were collected by the optical spectrum interrogator (si255 based on HYPERION platform; Micron Optics Inc.) at the sampling frequency of 100 Hz and the Instron machine output at the sampling frequency of 100 Hz.

The hysteresis errors (h_err_) of each test were calculated by computing the wavelength change difference between the ascending and the descending phases at the same input mε (${\Delta\lambda_{B}}^{a}-{\Delta\lambda_{B}}^{d}$) for each one of the twenty cycles and by finding the maximum $\Delta\lambda_{B}$value (${\Delta\lambda_{B}}^{\max}$ ) of the ascending phase of each cycle. The maximum difference of wavelength change was used to compute the hysteresis error for each cycle as follows:

$$h_{err}= \frac{\left( {\Delta\lambda_{B}}^{a}-{\Delta\lambda_{B}}^{d} \right)^{max}}{{\Delta\lambda_{B}}^{max}}\cdot100$$

Finally, the overall $h_{\mathrm{err}}$ for each velocity was estimated as the mean of the twenty $h_{\mathrm{err}}$ values. This data analysis was repeated for all the four FBGs, finding the average $h_{\mathrm{err}}$ value showed in Table 2. As expected, $h_{\mathrm{err}}$ value increases with the velocity.


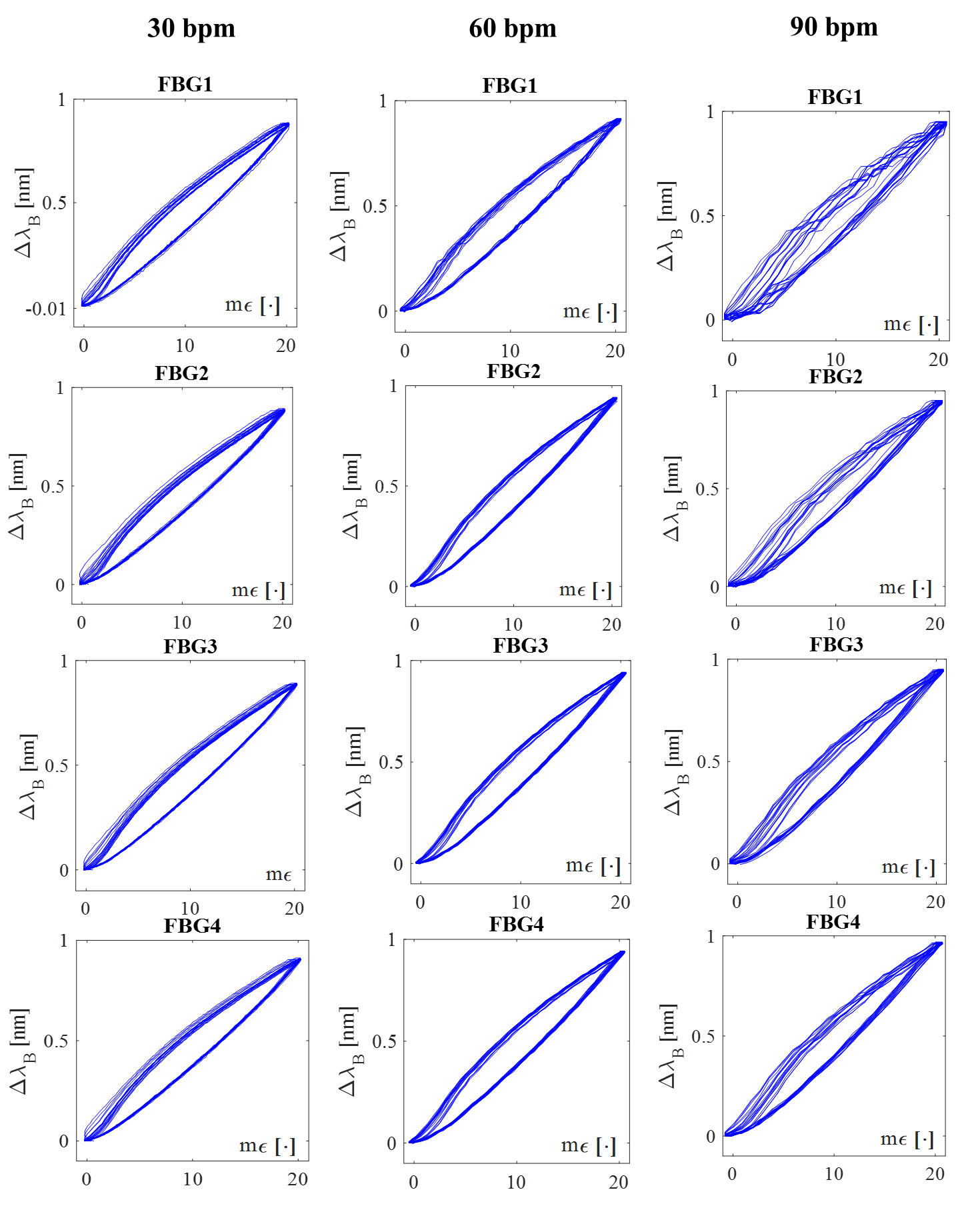


**Supplementary Figure 4**. The hysteresis loops obtained for FBG4 during velocities mimicking 30 bpm, 60 bpm, and 90 bpm.

**Supplementary Table 2**. The h_err_ values

|  | h_err_ [%] | | |
| --- | --- | --- | --- |
|  | **30 bpm (0.5 Hz)** | **60 bpm (1 Hz)** | **90 bpm (1.5 Hz)** |
| FBG 1 | 22.9 | 26.9 | 30.4 |
| FBG 2 | 22.6 | 26.8 | 28.4 |
| FBG 3 | 22.9 | 27.1 | 29.4 |
| FBG 4 | 22.6 | 26.6 | 28.9 |

# Sensor Placement

The wearable system was placed on three positions as shown in the following figure (partially available from https://www.dimensions.com):

- Position 1: horizontally in correspondence of the left side of the sternum from the xiphoid process to the heart apex.
- Position 2: vertically from the xyphoid process to the umbilicus.
- Position 3: horizontally above the umbilicus.


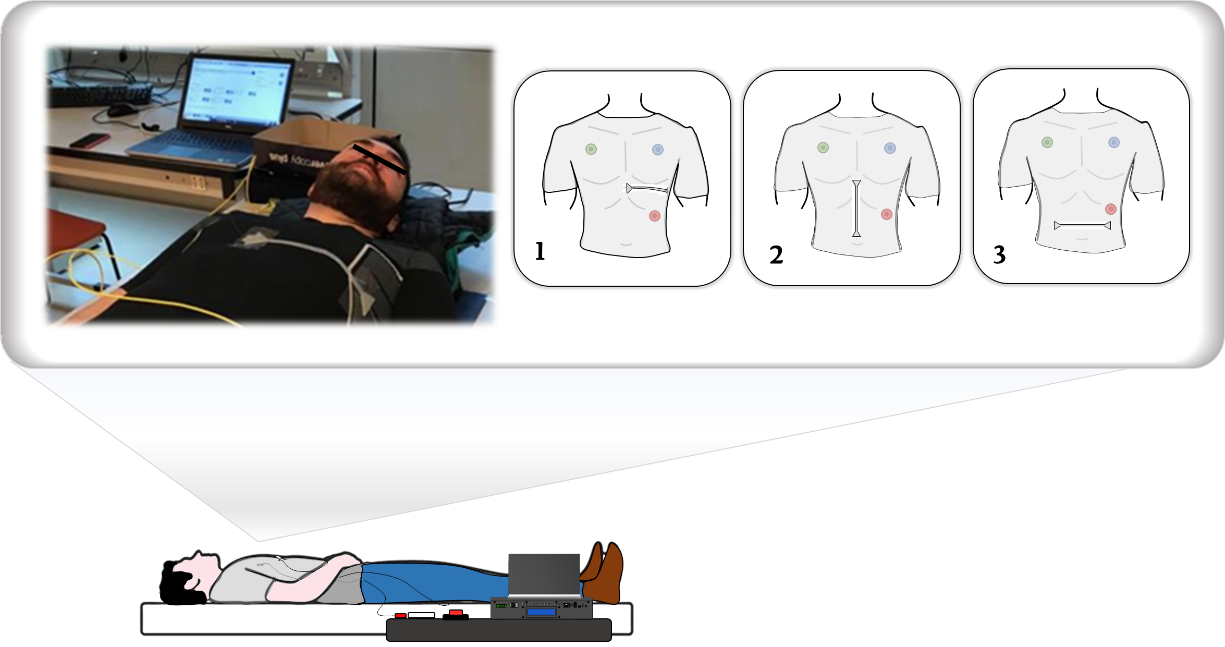


**Supplementary Figure 5**. The sensor positioning while volunteer is supine. The flexible matrix is attached on an elastic T-shirt using a medical tape. The sensor is placed first in position 1, then removed and placed on position 2, and lastly on position 3. The same procedure was cyclically repeated three times.

# Data analysis and Results

- 1. Data Analysis
     1. *Synchronization*

The soft wearable system was taped to Position 1, Position 2, and Position3 cyclically. During each test, each subject was invited to hold his breath for ~ 20 s after performing a few number of breaths. The FBGs output and the reference one (from the ECG) were synchronized using a trigger signal at the beginning of the apnea stage (black cross in the Fig below), the subject pressed a push button with a pull-up resistor close to the soft sensor to generate a trigger signal necessary for data synchronization.

*
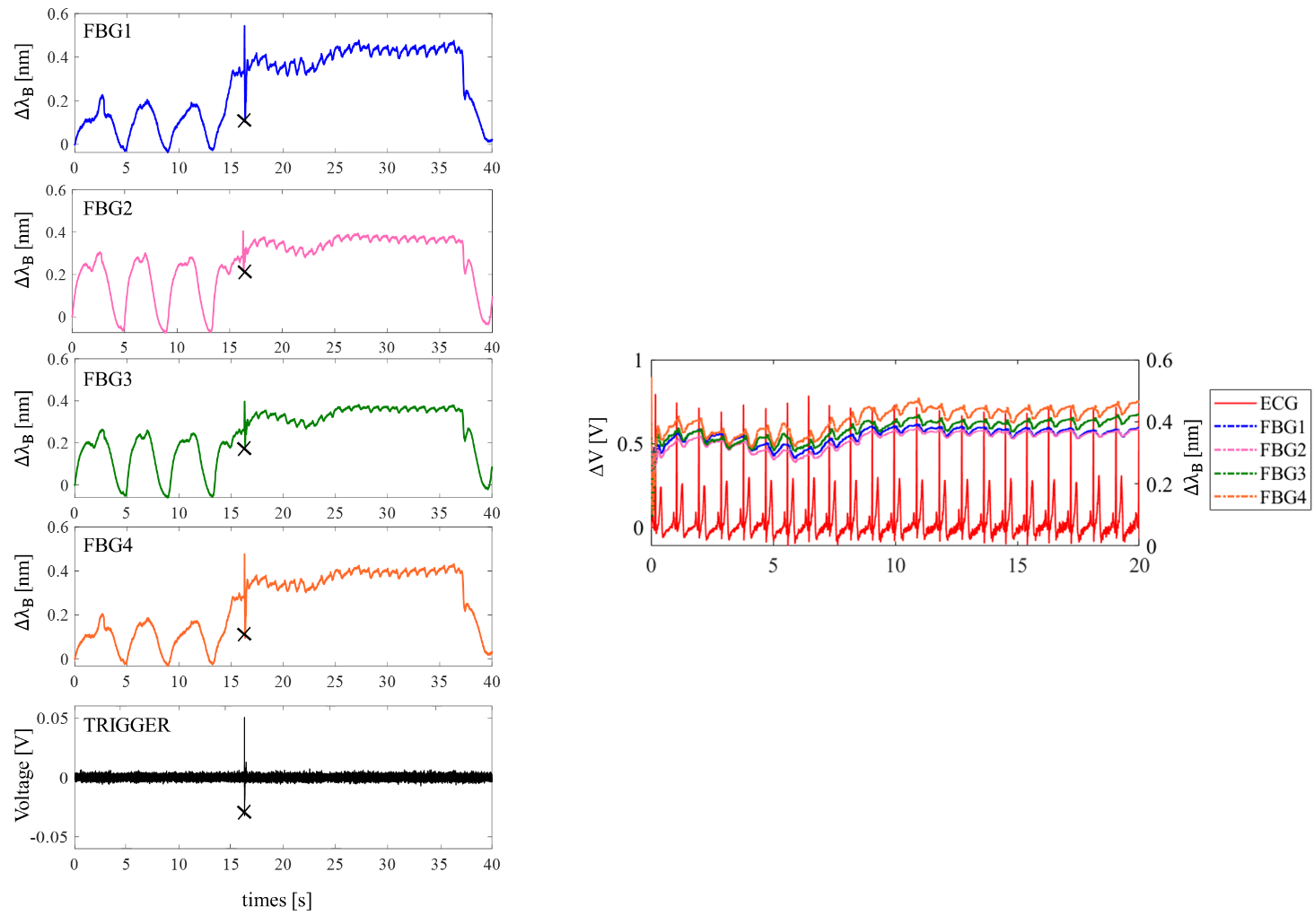
*

**Supplementary Figure 6**. On the left, the raw data gathered by FBG1 (blue line), FBG2 (magenta line); FBG3 (green line), FBG4 (orange line), and the trigger signal (black line), respectively; on the right, all the signals (FBG1, FBG2, FBG3, FBG4, and ECG) cut from the spikes (highlighted by the black cross) to the end of the apnea stages. The ECG is cut starting from the TRIGGER since both these signals are synchronously collected using the DAQ.

- - 1. *
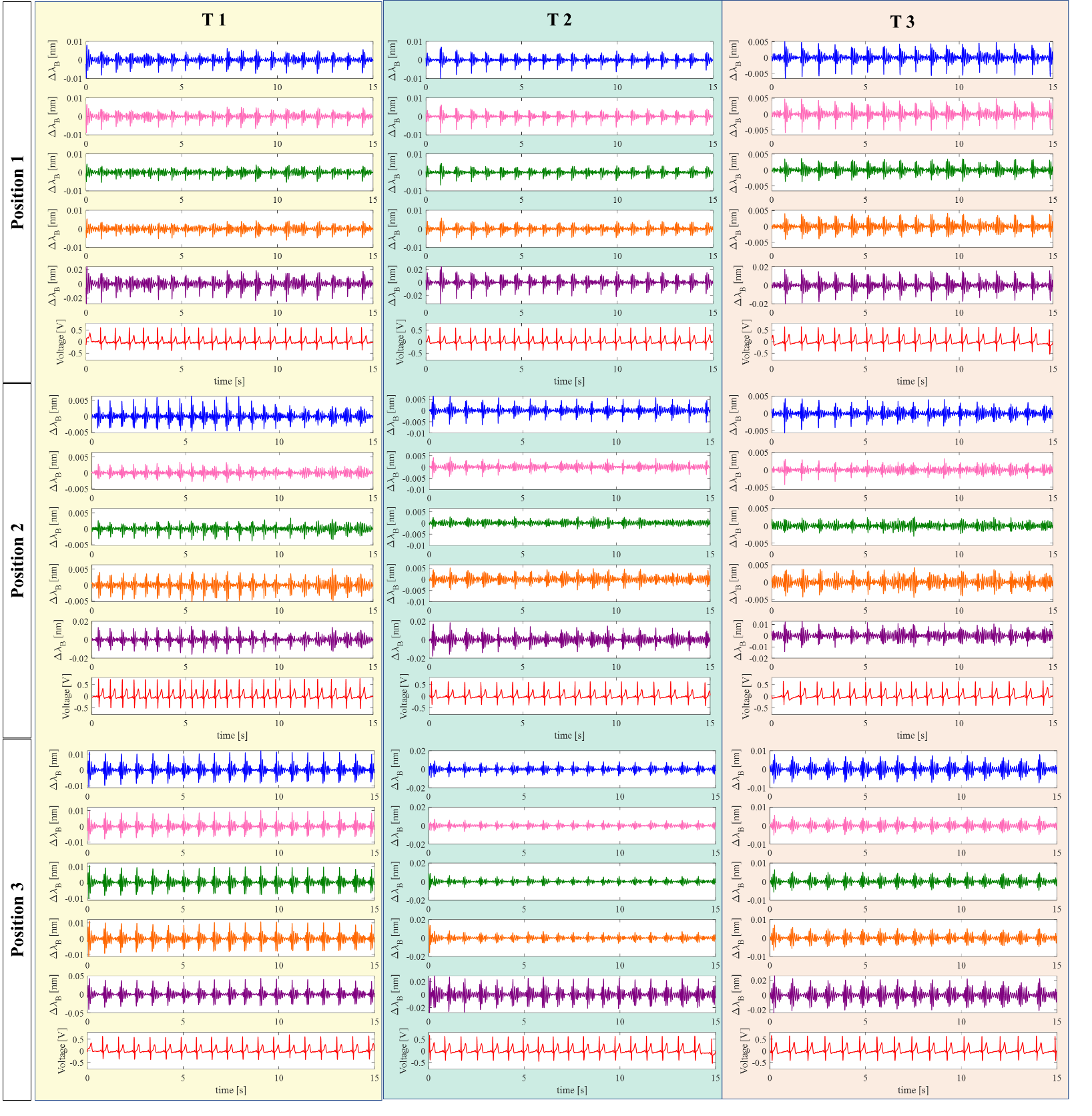
Filtering stage to extract SCG signals from FBG output*

**Supplementary Figure 7**. For Position 1, Position 2, and Position 3: the reference ECG signal (first row – red line) and the wearable system outputs: FBG1 (second row – blue line), FBG2 (third row – magenta line), FBG3 (forth row – green line), FBG4 (fifth row – orange line) and FBGsum (sixth row – purple line). In each Position, the first, second and third columns refer to the data gathered during T1, T2, and T3.

*4.1.3 SCG envelope and 2^nd^ filtering stage for findpeaks function*

**
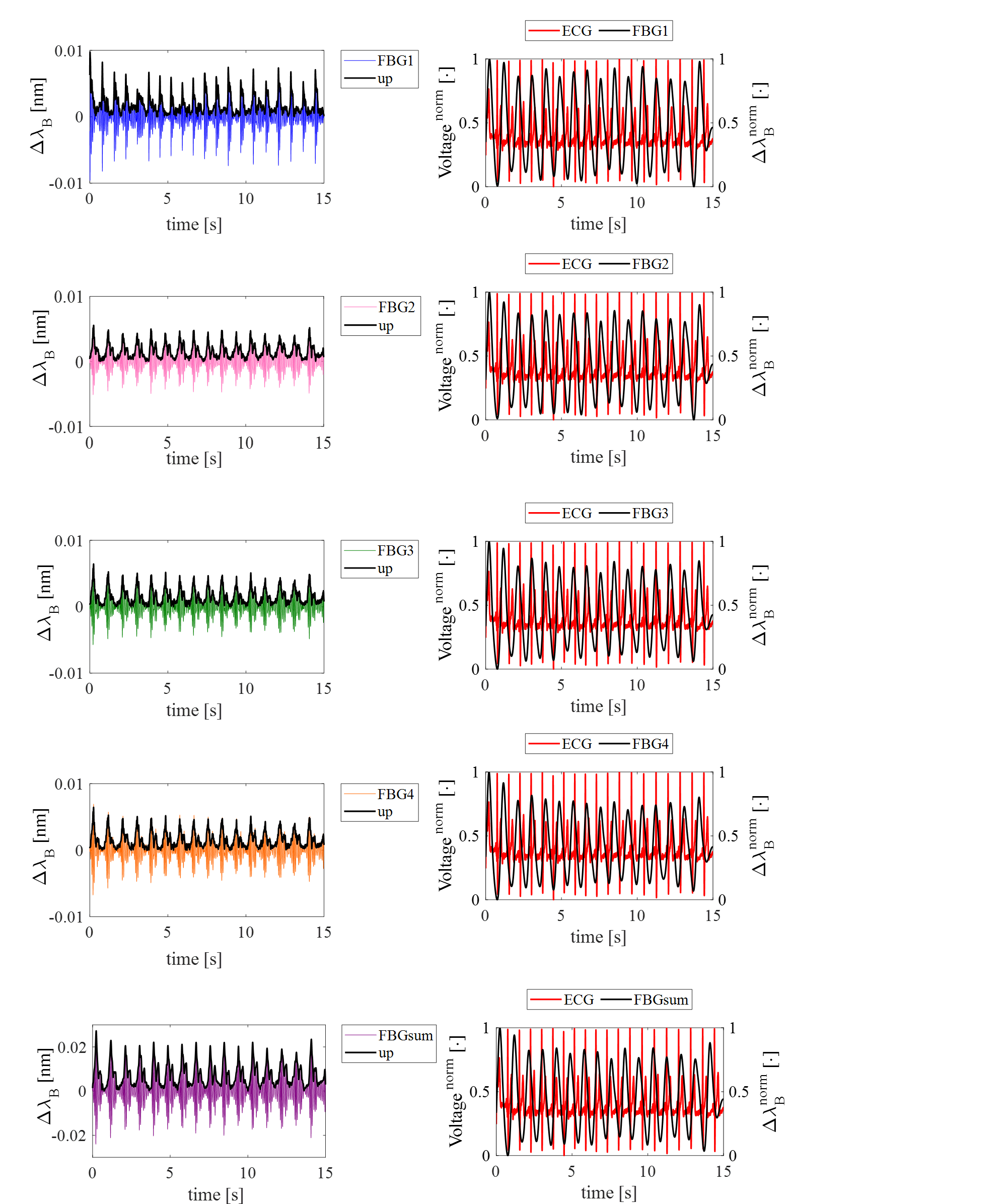
**

**Supplementary Figure 8**. On the left, the SCG signals (blue, magenta, green, orange and purple lines) with the envelope (black signal) and on the right the filtered SCG up envelope (black line) and the reference ECG signal (red line), for FBG1, FBG2, FBG3, FBG4, FBGsum respectively from top to bottom.


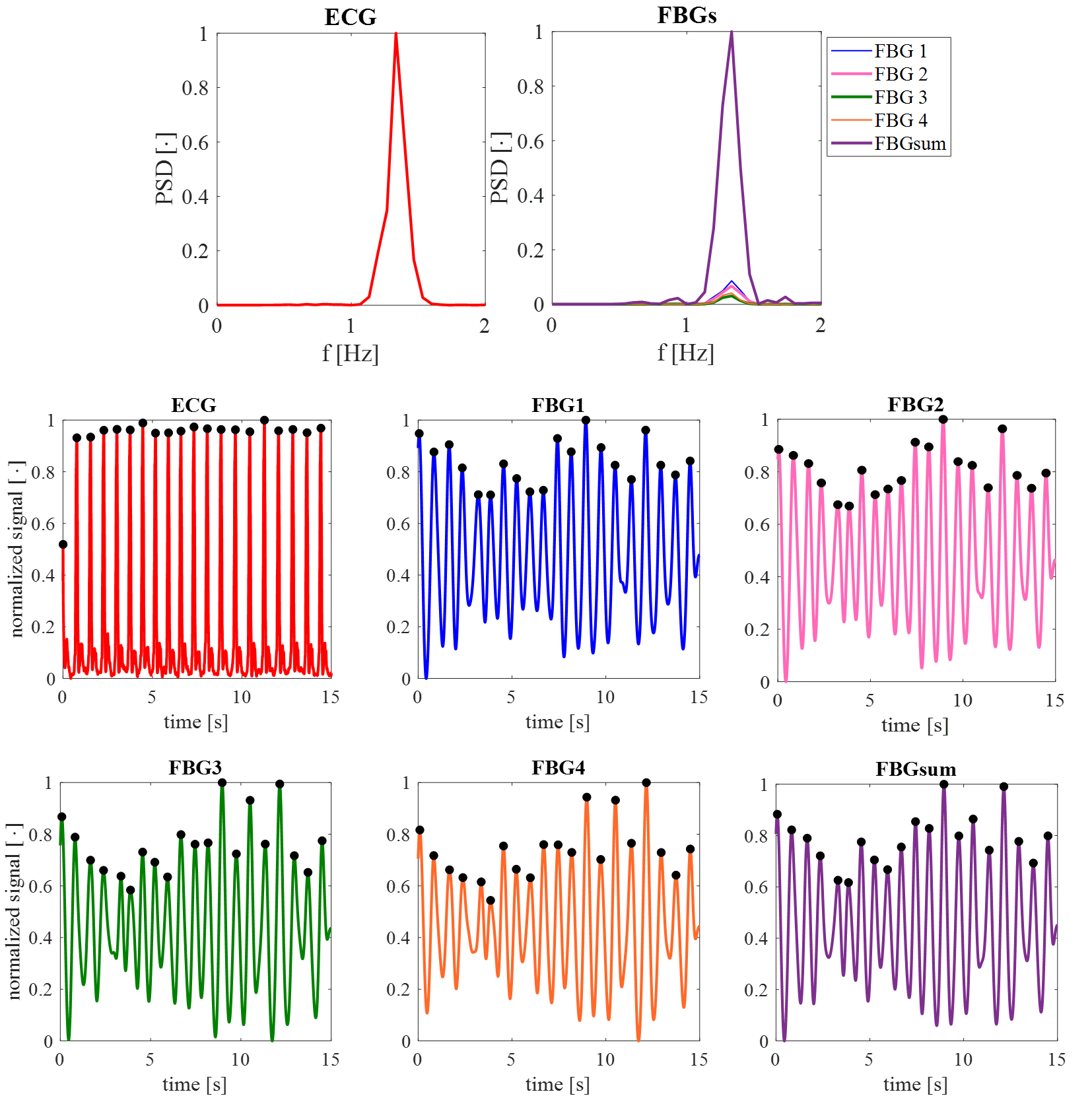


b

a

**Supplementary Figure 9**. a The Welch’s PSD. This analysis is necessary to define the fundamental frequency (i.e., the frequency at which the maximum of the PSD is located) for the ECG, FBG1, FBG2, FBG3, FBG4, and FBGsum signals. The PSD is evaluated using pwelch function in MATLAB environments on the SCG filtered envelope and the filtered ECG trace. B Peaks Detection. The frequencies from the PSD are used to define the minimum distances (MinPeakDistance) at which findpeaks function should detect peaks on the signals (please see METHODS section in the paper).

- 1. Results

A total of 21 HR values per each position have been estimated. Table 4-1 listed the mean HR values estimated from S1, S2, S3, S4, S5, S6, S7 in Position1, Position2, Position 3 respectively for FBG1, FBG2, FBG3, FBG4, FBGsum and the reference ECG sensor.

**Supplementary Table 3**. The mean HR values estimated in Position 1

|  | **Position 1 – HR [bpm]** | | | | | | | | | | | | | | | | | | |
| --- | --- | --- | --- | --- | --- | --- | --- | --- | --- | --- | --- | --- | --- | --- | --- | --- | --- | --- | --- |
|  | **FBG1** | | | **FBG2** | | | **FBG3** | | | **FBG4** | | | **FBGsum** | | | | **ECG** | | |
|  | **T1** | **T2** | **T3** | **T1** | **T2** | **T3** | **T1** | **T2** | **T3** | **T1** | **T2** | **T3** | **T1** | **T2** | **T3** | **T1** | | **T2** | **T3** |
| **S1** | 89.58 | 83.95 | 78.34 | 89.58 | 83.95 | 78.41 | 93.51 | 87.55 | 78.83 | 93.59 | 73.03 | 79.14 | 89.58 | 83.94 | 78.34 | 89.70 | | 83.99 | 78.36 |
| **S2** | 74.01 | 78.21 | 74.52 | 73.37 | 77.14 | 74.51 | 74.56 | 76.91 | 80.30 | 75.02 | 77.48 | 77.95 | 73.87 | 77.08 | 75.93 | 72.88 | | 72.46 | 70.02 |
| **S3** | 60.12 | 56.79 | 54.25 | 58.52 | 56.96 | 55.00 | 58.88 | 57.82 | 54.15 | 58.88 | 59.80 | 55.35 | 58.88 | 56.25 | 54.32 | 58.56 | | 56.10 | 53.45 |
| **S4** | 67.41 | 76.41 | 68.12 | 67.41 | 75.47 | 68.51 | 67.88 | 69.56 | 69.83 | 67.33 | 69.36 | 69.99 | 67.40 | 69.92 | 68.19 | 66.84 | | 69.53 | 67.75 |
| **S5** | 60.19 | 53.26 | 52.27 | 60.17 | 53.26 | 52.28 | 60.64 | 53.27 | 52.28 | 60.24 | 53.25 | 52.27 | 60.18 | 53.26 | 52.27 | 60.16 | | 53.26 | 52.31 |
| **S6** | 54.25 | 55.31 | 54.78 | 54.06 | 55.28 | 54.79 | 54.27 | 49.66 | 54.79 | 54.27 | 55.25 | 54.78 | 54.12 | 55.30 | 54.78 | 53.87 | | 53.34 | 54.73 |
| **S7** | 79.18 | 78.06 | 68.09 | 79.18 | 78.08 | 68.09 | 79.19 | 78.17 | 68.08 | 79.33 | 78.29 | 68.47 | 79.18 | 78.10 | 68.09 | 79.28 | | 77.97 | 68.13 |

|  | **Position 2 – HR [bpm]** | | | | | | | | | | | | | | | | | |
| --- | --- | --- | --- | --- | --- | --- | --- | --- | --- | --- | --- | --- | --- | --- | --- | --- | --- | --- |
|  | **FBG1** | | | **FBG2** | | | **FBG3** | | | **FBG4** | | | **FBGsum** | | | **ECG** | | |
|  | **T1** | **T2** | **T3** | **T1** | **T2** | **T3** | **T1** | **T2** | **T3** | **T1** | **T2** | **T3** | **T1** | **T2** | **T3** | **T1** | **T2** | **T3** |
| **S1** | 90.53 | 84.41 | 80.50 | 88,10 | 96.34 | 79.11 | 89.77 | 85.93 | 82.70 | 89.68 | 89.49 | 84,41 | 89,49 | 84,41 | 96,34 | 87,54 | 82,80 | 77,60 |
| **S2** | 70.46 | 74.88 | 74.42 | 71.30 | 75.96 | 73.41 | 71.83 | 77.12 | 78.97 | 72.73 | 68.54 | 74.88 | 68.54 | 74.88 | 75.96 | 70.35 | 70.34 | 73.37 |
| **S3** | 60.88 | 52.03 | 53.54 | 65.53 | 52.67 | 53.73 | 59.47 | 53.68 | 53.91 | 59.93 | 60.84 | 52.03 | 60.84 | 52.03 | 52.67 | 57.07 | 51.43 | 53.85 |
| **S4** | 75.85 | 71.00 | 84.17 | 73.79 | 72.08 | 76.94 | 65.22 | 68.68 | 72.26 | 65.09 | 66.47 | 71.00 | 66.47 | 71.00 | 72.08 | 63.39 | 68.37 | 72.05 |
| **S5** | 64.53 | 57.18 | 55.50 | 64.25 | 55.52 | 55.38 | 61.79 | 54.50 | 63.56 | 56.08 | 55.97 | 57.18 | 55.97 | 57.18 | 55.52 | 57.61 | 53.45 | 54.37 |
| **S6** | 55.03 | 54.47 | 63.99 | 58.43 | 60.69 | 66.47 | 56.70 | 57.49 | 59.66 | 56.22 | 55.88 | 54.47 | 55.88 | 54.47 | 60.69 | 52.34 | 54.18 | 56.61 |
| **S7** | 90.61 | 69.91 | 69.45 | 93.53 | 70.13 | 71.08 | 91.11 | 70.68 | 72.99 | 90.78 | 90.60 | 69.91 | 90.60 | 69.91 | 70.13 | 91.00 | 69.93 | 69.43 |
|  | **Position 3 – HR [bpm]** | | | | | | | | | | | | | | | | | |
|  | **FBG1** | | | **FBG2** | | | **FBG3** | | | **FBG4** | | | **FBGsum** | | | **ECG** | | |
|  | **T1** | **T2** | **T3** | **T1** | **T2** | **T3** | **T1** | **T2** | **T3** | **T1** | **T2** | **T3** | **T1** | **T2** | **T3** | **T1** | **T2** | **T3** |
| **S1** | 86.10 | 88.27 | 85.64 | 88.27 | 85.64 | 86.06 | 85.64 | 86.06 | 87.91 | 86.06 | 87.91 | 83.19 | 87.91 | 83.19 | 84.18 | 85.20 | 84.03 | 77.83 |
| **S2** | 79.48 | 78.25 | 83.56 | 78.25 | 83.56 | 79.36 | 83.56 | 79.36 | 79.64 | 79.36 | 79.64 | 68.12 | 79.64 | 68.12 | 64.95 | 74.17 | 69.27 | 69.59 |
| **S3** | 125.6 | 51.02 | 53.65 | 118.0 | 51.02 | 60.17 | 122.9 | 51.03 | 57.91 | 128.6 | 51.03 | 55.88 | 122.9 | 51.3 | 60.50 | 60.56 | 50.88 | 56.90 |
| **S4** | 70.30 | 70.29 | 70.34 | 70.29 | 70.34 | 68.92 | 70.34 | 68.92 | 70.32 | 68.92 | 70.32 | 70.06 | 70.32 | 70.06 | 70.29 | 68.56 | 70.05 | 68.20 |
| **S5** | 59.33 | 58.47 | 57.67 | 58.47 | 57.67 | 58.31 | 57.67 | 58.31 | 56.92 | 58.31 | 56.92 | 53.97 | 56.92 | 53.97 | 53.99 | 56.16 | 53.92 | 55.68 |
| **S6** | 56.73 | 60.40 | 60.48 | 60.40 | 60.48 | 51.60 | 60.48 | 51.60 | 56.67 | 51.60 | 56.67 | 63.21 | 56.67 | 63.21 | 56.11 | 53.56 | 57.35 | 55.82 |
| **S7** | 73.26 | 73.26 | 73.47 | 73.26 | 73.47 | 73.42 | 73.47 | 73.42 | 73.31 | 73.42 | 73.31 | 73.01 | 73.31 | 73.01 | 73.73 | 73.33 | 73.11 | 64.82 |

The differences in terms of bpm between the HR values estimated by the FBGs and the reference ones have been assessed by calculating the mean absolute error (MAE) as:

MAE [bpm] = $\frac{1}{n}\sum_{i=1}^{n} \left| {HR}_{i}^{wearable}- {HR}_{i}^{reference} \right|$

All the MAE values are listed in Table 4-2, according to Position 1, Position 2, and Position 3, respectively.

**Supplementary Table 4**. The MAE values for each FBG during each test grouped for position.

|  | **Position 1 - MAE [bpm]** | | | | | | | | | | | | | | |
| --- | --- | --- | --- | --- | --- | --- | --- | --- | --- | --- | --- | --- | --- | --- | --- |
|  | **FBG1** | | | **FBG2** | | | **FBG3** | | | **FBG4** | | | **FBGsum** | | |
|  | **T1** | **T2** | **T3** | **T1** | **T2** | **T3** | **T1** | **T2** | **T3** | **T1** | **T2** | **T3** | **T1** | **T2** | **T3** |
| **S1** | 0.12 | 0.03 | 0.02 | 0.12 | 0.05 | 0.56 | 3.81 | 3.56 | 0.47 | 3.89 | 10.96 | 0.78 | 0.12 | 0.05 | 0.02 |
| **S2** | 1.14 | 5.75 | 4.49 | 0.49 | 4.49 | 0.95 | 1.68 | 4.45 | 10.28 | 2.15 | 5.02 | 7.93 | 0.99 | 4.62 | 5.90 |
| **S3** | 1.56 | 0.70 | 0.80 | 0.04 | 1.55 | 8.46 | 0.32 | 1.72 | 0.70 | 0.32 | 3.70 | 1.90 | 0.32 | 0.16 | 0.87 |
| **S4** | 0.57 | 6.87 | 0.38 | 0.57 | 0.77 | 10.40 | 1.04 | 0.03 | 2.08 | 0.49 | 0.17 | 2.24 | 0.56 | 0.39 | 0.45 |
| **S5** | 0.03 | 0.01 | 0.03 | 0.01 | 0.03 | 6.63 | 0.48 | 0.01 | 0.03 | 0.07 | 0.01 | 0.04 | 0.02 | 0.00 | 0.04 |
| **S6** | 0.39 | 1.97 | 0.05 | 0.20 | 0.06 | 6.09 | 0.40 | 3.68 | 0.06 | 0.40 | 1.91 | 0.05 | 0.25 | 1.95 | 0.05 |
| **S7** | 0.10 | 0.09 | 0.04 | 0.10 | 0.04 | 2.53 | 0.09 | 0.20 | 0.05 | 0.06 | 0.33 | 0.34 | 0.09 | 0.13 | 0.04 |
|  | **Position 2 - MAE [bpm]** | | | | | | | | | | | | | | |
|  | **FBG1** | | | **FBG2** | | | **FBG3** | | | **FBG4** | | | **FBGsum** | | |
|  | **T1** | **T2** | **T3** | **T1** | **T2** | **T3** | **T1** | **T2** | **T3** | **T1** | **T2** | **T3** | **T1** | **T2** | **T3** |
| **S1** | 3.00 | 1.61 | 2.90 | 0.56 | 13.54 | 1.51 | 2.23 | 3.13 | 5.10 | 2.15 | 6.58 | 4.64 | 1.95 | 11.98 | 4.69 |
| **S2** | 0.10 | 4.54 | 1.06 | 0.95 | 5.63 | 0.05 | 1.48 | 6.79 | 5.60 | 2.38 | 7.24 | 11.38 | 1.81 | 6.44 | 0.71 |
| **S3** | 3.81 | 0.59 | 0.30 | 8.46 | 1.24 | 0.12 | 2.40 | 2.24 | 0.06 | 2.86 | 1.69 | 0.74 | 3.76 | 2.23 | 0.31 |
| **S4** | 12.46 | 2.63 | 12.12 | 10.40 | 3.71 | 4.89 | 1.83 | 0.31 | 0.21 | 1.70 | 0.70 | 0.22 | 3.08 | 1.99 | 0.36 |
| **S5** | 6.92 | 3.73 | 1.13 | 6.63 | 2.07 | 1.01 | 4.18 | 1.05 | 9.19 | 1.53 | 1.63 | 2.85 | 1.65 | 0.01 | 0.10 |
| **S6** | 2.69 | 0.29 | 7.38 | 6.09 | 6.51 | 9.86 | 4.36 | 3.31 | 3.05 | 3.88 | 0.06 | 4.19 | 3.54 | 5.32 | 1.53 |
| **S7** | 0.39 | 0.02 | 0.02 | 2.53 | 0.20 | 1.66 | 0.11 | 0.75 | 3.57 | 0.22 | 0.11 | 1.75 | 0.40 | 0.15 | 3.66 |
|  | **Position 2 - MAE [bpm]** | | | | | | | | | | | | | | |
|  | **FBG1** | | | **FBG2** | | | **FBG3** | | | **FBG4** | | | **FBGsum** | | |
|  | **T1** | **T2** | **T3** | **T1** | **T2** | **T3** | **T1** | **T2** | **T3** | **T1** | **T2** | **T3** | **T1** | **T2** | **T3** |
| **S1** | 0.90 | 0.84 | 0.02 | 3.07 | 0.15 | 0.31 | 0.44 | 3.68 | 0.06 | 0.86 | 4.64 | 0.05 | 2.72 | 0.15 | 0.00 |
| **S2** | 5.31 | 1.15 | 4.95 | 4.08 | 4.32 | 5.02 | 9.39 | 2.64 | 6.37 | 5.19 | 5.70 | 5.91 | 5.47 | 6.89 | 5.41 |
| **S3** | 65.0 | 0.13 | 3.25 | 57.5 | 0.14 | 3.27 | 62.4 | 0.15 | 1.01 | 68.0 | 0.14 | 1.10 | 62.4 | 0.15 | 3.60 |
| **S4** | 1.75 | 0.00 | 0.30 | 1.74 | 0.24 | 0.34 | 1.78 | 0.01 | 0.08 | 0.36 | 0.31 | 0.13 | 1.77 | 0.00 | 0.14 |
| **S5** | 3.17 | 0.04 | 1.02 | 2.31 | 0.06 | 3.14 | 1.51 | 0.14 | 0.85 | 2.15 | 0.03 | 0.86 | 0.76 | 0.03 | 0.22 |
| **S6** | 3.16 | 5.86 | 1.37 | 6.84 | 1.24 | 5.42 | 6.91 | 6.56 | 0.03 | 1.96 | 7.13 | 14.33 | 3.11 | 5.51 | 1.27 |
| **S7** | 0.07 | 0.10 | 0.26 | 0.07 | 0.61 | 0.26 | 0.14 | 0.16 | 0.36 | 0.10 | 0.02 | 0.37 | 0.02 | 0.20 | 0.32 |


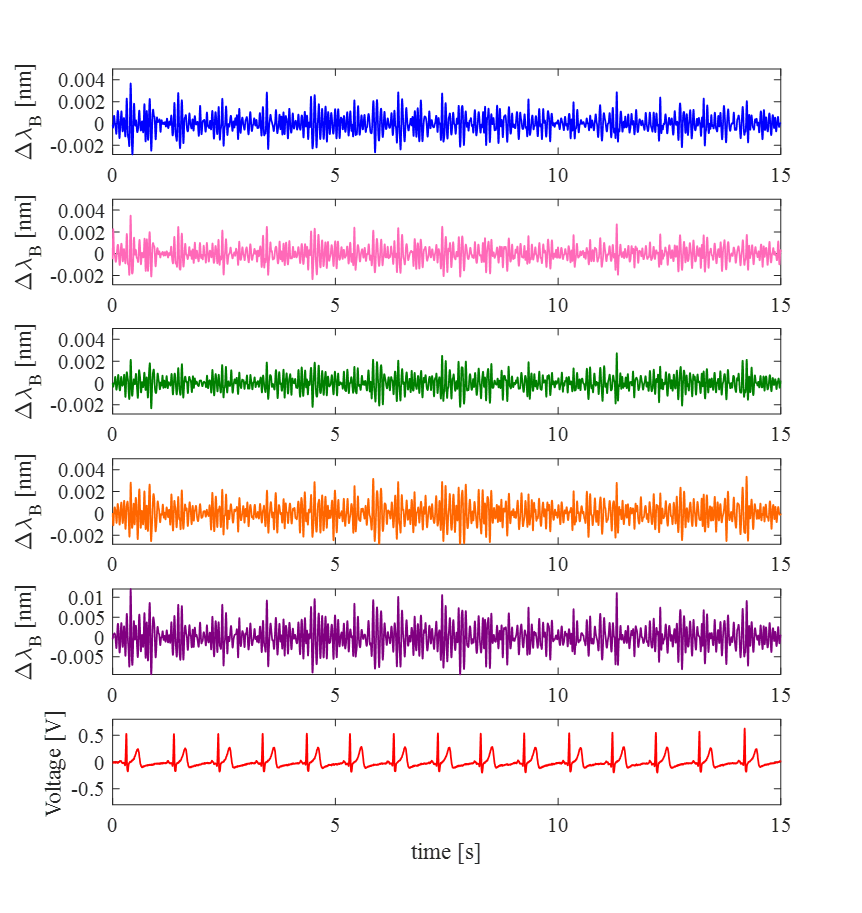


**Supplementary Figure 10**. The SCG signals obtained by FBG1, FBG2, FBG3, FBG4, FBGsum (blue, magenta, green, orange, purple lines, respectively) with the reference ECG signal (red line) from S3 in Position 3 during T1.

Results of the analyses (i.e., Position and Single-sensor vs. Multi-sensor analyses) carried out to investigate the SWS performance are listed below.

**Supplementary Table 5**. The MAE values found considering Position 1, Position 2, and Position 3, respectively.

|  |  | **MAE [bpm]** | | | | |
| --- | --- | --- | --- | --- | --- | --- |
|  |  | **FBG1** | **FBG2** | **FBG3** | **FBG4** | **FBGsum** |
| ***Position analysis*** | **Position 1** | 1.20 | 2.10 | 1.67 | 2.04 | 0.81 |
|  | **Position 2** | 3.22 | 4.17 | 2.90 | 2.63 | 2.65 |
|  | **Position 3** | 1.68 | 2.13 | 2.11 | 2.57 | 1.89 |
| ***Single-sensor vs. Multi-sensor analysis*** | **Comprehensive Evaluation** | 2.04 | 2.81 | 2.23 | 2.41 | 1.78 |
